# Inositol 1,4,5-trisphosphate receptor type 1 autoantibodies in paraneoplastic and non-paraneoplastic peripheral neuropathy

**DOI:** 10.1186/s12974-016-0737-x

**Published:** 2016-10-24

**Authors:** Sven Jarius, Marius Ringelstein, Jürgen Haas, Irina I. Serysheva, Lars Komorowski, Kai Fechner, Klaus-Peter Wandinger, Philipp Albrecht, Harald Hefter, Andreas Moser, Eva Neuen-Jacob, Hans-Peter Hartung, Brigitte Wildemann, Orhan Aktas

**Affiliations:** 1 Molecular Neuroimmunology Group, Department of Neurology, University Hospital Heidelberg, Otto Meyerhof Center, Im Neuenheimer Feld 350, 69120 Heidelberg, Germany; 2 Department of Neurology, Medical Faculty, Heinrich Heine University, Moorenstraße 5, 40225 Düsseldorf, Germany; 3 Department of Biochemistry and Molecular Biology, The University of Texas Medical School at Houston, 6431 Fannin Street, Houston, TX 77030 USA; 4 Institute of Experimental Immunology, affiliated to Euroimmun AG, Seekamp 31, 23560 Lübeck, Germany; 5 Department of Neurology, University of Schleswig Holstein, Ratzeburger Allee 160, 23538 Lübeck, Germany; 6 Department of Neuropathology, Medical Faculty, Heinrich Heine University, Moorenstraße 5, 40225 Düsseldorf, Germany

**Keywords:** Anti-neuronal autoantibodies, Inositol 1,4,5-trisphosphate type 1 receptor antibodies, Paraneoplastic neurological syndrome, Guillain-Barré syndrome (GBS)﻿, Acute sensorimotor polyradiculopathy, Polyneuropathy, Neuritis, Autonomic neuropathy, Lung c﻿ancer, Adenocarcinoma of the lung, Multiple myeloma, Pain, Cranial nerve palsy, Facial nerve paralysis, Autoimmune cerebellar ataxia, Neuropathology

## Abstract

**Background:**

Recently, we described a novel autoantibody, anti-Sj/ITPR1-IgG, that targets the inositol 1,4,5-trisphosphate receptor type 1 (ITPR1) in patients with cerebellar ataxia. However, ITPR1 is expressed not only by Purkinje cells but also in the anterior horn of the spinal cord, in the substantia gelatinosa and in the motor, sensory (including the dorsal root ganglia) and autonomic peripheral nervous system, suggesting that the clinical spectrum associated with autoimmunity to ITPR1 may be broader than initially thought. Here we report on serum autoantibodies to ITPR1 (up to 1:15,000) in three patients with (radiculo)polyneuropathy, which in two cases was associated with cancer (ITPR1-expressing adenocarcinoma of the lung, multiple myeloma), suggesting a paraneoplastic aetiology.

**Methods:**

Serological and other immunological studies, and retrospective analysis of patient records.

**Results:**

The clinical findings comprised motor, sensory (including severe pain) and autonomic symptoms. While one patient presented with subacute symptoms mimicking Guillain-Barré syndrome (GBS), the symptoms progressed slowly in two other patients. Electrophysiology revealed delayed F waves; a decrease in motor and sensory action potentials and conduction velocities; delayed motor latencies; signs of denervation, indicating sensorimotor radiculopolyneuropathy of the mixed type; and no conduction blocks. ITPR1-IgG belonged to the complement-activating IgG1 subclass in the severely affected patient but exclusively to the IgG2 subclass in the two more mildly affected patients. Cerebrospinal fluid ITPR1-IgG was found to be of predominantly extrathecal origin. A ^3^H-thymidine-based proliferation assay confirmed the presence of ITPR1-reactive lymphocytes among peripheral blood mononuclear cells (PBMCs). Immunophenotypic profiling of PBMCs protein demonstrated predominant proliferation of B cells, CD4 T cells and CD8 memory T cells following stimulation with purified ITPR1 protein. Patient ITPR1-IgG bound both to peripheral nervous tissue and to lung tumour tissue. A nerve biopsy showed lymphocyte infiltration (including cytotoxic CD8 cells), oedema, marked axonal loss and myelin-positive macrophages, indicating florid inflammation. ITPR1-IgG serum titres declined following tumour removal, paralleled by clinical stabilization.

**Conclusions:**

Our findings expand the spectrum of clinical syndromes associated with ITPR1-IgG and suggest that autoimmunity to ITPR1 may underlie peripheral nervous system diseases (including GBS) in some patients and may be of paraneoplastic origin in a subset of cases.

**Electronic supplementary material:**

The online version of this article (doi:10.1186/s12974-016-0737-x) contains supplementary material.

## Background

Recently, we described a new autoantibody (Ab) reactivity in patients with autoimmune cerebellar ataxia (ACA) which targets the inositol 1,4,5-trisphosphate receptor type 1 (ITPR1; also termed IP3RI) [1]. In the adult brain, ITPR1 is strongly expressed in Purkinje cells (PC) of the cerebellum, in hippocampal neurons (particularly in CA1 pyramidal cells) [2–4], and in the cerebral cortex (most pronounced in pyramidal layer V and in non-pyramidal layer II) [2, 3].

However, expression of ITPR1 has been found also in neuronal and glial cells (probably oligodendrocytes) in the posterior and anterior horn of the spinal cord [2, 3] and in neurons in the sensory dorsal root ganglia (DRG), the trigeminal ganglia and the sympathetic ganglia [2]. Moreover, ITPR1 has been implicated in nociception, with dysregulation of ITPR1 inhibition in the DRG leading to allodynia and hyperalgesia in mice [5]; ITPR1 is also expressed at a high level in the nociceptive substantia gelatinosa [3]. Here we report on three anti-ITPR1-positive patients who developed symptoms compatible with damage to the second motor neuron and/or first sensory neuron, with additional involvement of the autonomic nervous system and severe neurogenic pain in one of them. Of particular note, ITPR1 autoimmunity was associated with lung cancer in one of our patients and with multiple myeloma in another, suggesting a paraneoplastic aetiology.

In addition, we immunologically characterized the ITPR1-Ab-related immune response by investigating (i.) the peripheral blood mononuclear cell (PBMC) phenotypic profile associated with ITPR1-IgG positivity and the phenotype alterations in the PBMC compartment following stimulation with ITPR1, (ii.) the origin of ITPR1-IgG in the cerebrospinal fluid (CSF), and (iii.) the ITPR1 immunoglobulin (Ig) class and subclass distribution. Furthermore, we studied the expression profiles of ITPR1 in the spinal cord, DRG and sural nerve. Finally, we provide evidence in favour of a paraneoplastic aetiology of ITPR1 autoimmunity by demonstrating both expression of ITPR1 by the patient’s lung tumour and binding of patient IgG to the tumour tissue.

## Methods

### Immunohistochemistry

Testing for ITPR1-IgG, ITPR1-IgM and ITPR1-IgA by immunohistochemistry (IHC) was performed as previously described [1]. Briefly, 4-μm cryosections of mouse, rat and primate cerebellum and primate spinal cord (Euroimmun, Lübeck, Germany) and 10-μm cryosections of rat DRG tissue (Zyagen, San Diego, CA) as well as cryosections from an adenocarcinoma of the lung removed from a ITPR1-IgG-positive patient (patient 1) were carefully thawed and subsequently treated with formalin for 3 min, CHAPS for 3 min and 10 % goat serum for 1 h. Sections were then incubated for 1 h with diluted patient and control serum and with CSF samples. Binding of patient serum and CSF IgG was visualized using a goat secondary antibody to human IgG labelled with Alexa Fluor® (AF) 488 and a goat secondary antibody to human IgG labelled with FITC (Euroimmun), respectively. Binding of a commercial rabbit anti-ITPR1 antibody (Dianova, Hamburg, Germany﻿) was detected using a goat anti-rabbit IgG AF568 antibody (Invitrogen, Karlsruhe, Germany). The rabbit anti-ITPR1 antibody was chosen because it recognizes an epitope that, according to UniProt data, shares no sequence homology with ITPR2 and ITPR3.

### ITPR1-Ig class and subclass analysis

For further evaluation of Ig classes and IgG subclasses, unconjugated sheep anti-human IgG antibodies specific for IgG subclasses 1 to 4 (The Binding Site, Schwetzingen, Germany) and fluorescein isothiocyanate (FITC)-labelled goat antibodies specific for human IgA and IgM were substituted for the FITC-labelled goat anti-human IgG antibody and an Alexa Fluor® 568-labelled donkey anti-sheep IgG secondary antibody (Invitrogen; absorbed against human IgG) was used to detect the IgG subclass-specific secondary antibodies.

### ITPR1-specific dot-blot assay

The presence of ITPR1-specific IgG was confirmed using an immunoblot assay employing purified native ITPR1 from rat brain as previously described [1]. Briefly, Protran BA79 nitrocellulose membranes (Whatman) were spotted with increasing dilutions of full-length ITPR1 purified from rat cerebellum [6] and of ARHGAP26 (Abnova) in 0.1 % bovine serum albumin (BSA). After drying, membranes were blocked with 5 % BSA in Tris-buffered saline (TBS) for 1 h at room temperature (RT), washed three times in TBS with 0.05 % Tween (TBS-T) and finally incubated with a 1:20 dilution of the patient serum in 0.1 % BSA/TBS-T for 1 h at RT. A donkey anti-human IgG antibody labelled with IRdye 700DX (Rockland) was used to detect bound IgG. Stripes were finally washed in TBS and analysed using an Odyssey™ fluorescence scanner (Licor, Lincoln, NE, USA) and Odyssey™ 2.0.40 application software (Licor). As controls, serum samples from healthy donors and ARHGAP26-IgG-positive patients [7–9] were tested in the same run.

### ITPR1-specific cell-based assay

Finally, specificity of the patients’ IgG response for ITPR1 was further confirmed using a cell-based assay (﻿CBA) as previously reported [1]. Briefly, murine full-length ITPR1 was expressed in human embryonic kidney (HEK) 293 cells. HEK293 cells were then grown on sterile cover glasses for 48 h and subsequently fixed with acetone. Cover glasses were then cut into millimetre-sized fragments (biochips), which were used as assay substrate. Biochips with mock-transfected HEK293 cells were used as control substrate. Both substrates were incubated with the patients’ serum samples or control samples in parallel. Bound IgG was detected using a goat secondary antibody to human IgG labelled with FITC (Euroimmun).

### ITPR1-specific antibody index

A paired CSF/serum sample obtained 27 days after onset of symptoms was available from patient 1. To assess the origin of CSF ITPR1, the ITPR1-specific antibody index (AI), AI_ITPR1_, was calculated [10–12]. Briefly, AI_ITPR1_ values were calculated as the ratio between the CSF/serum quotient for ITPR1-IgG, Q_ITPR1_, and the CSF/serum quotient for total IgG, Q_IgG_, i.e. AI_ITPR1_ = Q_ITPR1_/Q_IgG_. If ITPR1-IgG are produced intrathecally, Q_ITPR1_ would exceed Q_IgG_, resulting in AI_ITPR1_ values >1. Usually, values >1.5 are considered as evidence of intrathecal synthesis of antibodies to the respective antigen [13, 14]. However, if titres instead of concentrations are used to calculate the AI, a more conservative cut-off of 4 has been recommended [10]. Reiber’s empiric hyperbolic function Q_lim_ was applied to control for possible underestimation of intrathecal specific synthesis due to disturbances of the blood-CSF barrier function [15]:

$$ {\mathrm{Q}}_{lim\left(\mathrm{I}\mathrm{g}\mathrm{G}\right)}=0.93\sqrt{{\left({\mathrm{Q}}_{\mathrm{AIb}}\right)}^2+6\times {10}^{-6}}-1.7\times {10}^{-3} $$

In the case of Q_IgG_ > Q_lim(IgG)_, AI_ITPR1_ was calculated as the ratio of Q_ITPR1_ and Q_lim(IgG)_, i.e. AI_ITPR1_ = Q_ITPR1_/Q_lim(IgG)_. For assessment of the AI, serum samples were tested at dilutions of 1:10, 1:32, 1:100, 1:320, 1:1,000, 1:3,200 and 1:10,000. CSF samples were tested undiluted, at 1:10 dilution and, in addition, at dilutions that would indicate intrathecal production as defined by an ITPR1-AI of >4. The CSF/serum albumin ratio, Q_Alb_ = Alb_CSF[mg/l]_/Alb_serum[g/l]_, was used to assess the blood-CSF barrier function. The upper reference limit of Q_Alb_, Q_lim(Alb)_, was calculated as 4 + (*a*/15), with a representing the patient’s age [15]. Total IgG and albumin values were determined nephelometrically.

### Peripheral blood mononuclear cell phenotype profiling

PBMCs were isolated from peripheral blood from patient 1 and a healthy control by density gradient centrifugation using Ficoll-Hypaque (Biochrom AG, Berlin, Germany) and immediately subjected to multi-colour flow cytometric analysis. For B cell phenotyping, 10^6^ PBMCs were stained with fluorescent-dye-labelled monoclonal antibodies (mAbs) specific for CD20, CD27 and IgD. Stained cells were first gated for CD20^+^ (total B cells), followed by CD27/IgD dot-plot analysis identifying CD20^+^CD27^−^IgD^+^ naïve, CD20^+^CD27^+^IgD^+^ non-switched memory B cells, and CD20^+^CD27^+^IgD^−^ isotype class-switched memory B cells [16]. For detection of T lymphocytes, 10^6^ PBMCs were stained with fluorescent-dye-labelled mAbs specific for CD3, CD4, CD8 and CD45RO. Stained cells were first gated for CD3 (total T cells), followed by CD4/CD45RO and CD8/CD45RO dot-plot analysis to identify CD3^+^CD4^+^ T-helper cells, CD3^+^CD4^+^CD45^+^ memory T-helper cells, CD3^+^CD4^+^CD45RO^−^ naïve T-helper cells, CD3^+^CD8^+^ cytotoxic T cells, CD3^+^CD8^+^CD45^+^ memory cytotoxic T cells and CD3^+^CD8^+^CD45RO^−^ naïve cytotoxic T cells. All mAbs were purchased from BD Pharmingen (Heidelberg, Germany). Fluorescence-activated cell sorting (FACS) data acquisition was performed with a FACS Calibur™ cytometer and analysed with CellQuest™ software (BD Biosciences, Heidelberg, Germany).

### Blood cell proliferation assay

Mononuclear blood cells (2 × 10^5^/200 μl) from patient 1 (sample obtained prior to tumour therapy) and a healthy control donor were cultured in triplicate in U-bottom microtitre wells (TPP Techno Plastic Products AG, Trasadingen, Switzerland) in RPMI 1640 medium supplemented with 10 % heat-inactivated AB human serum (Sigma Chemie), 2 mM l-glutamine and 100 U/ml penicillin-streptomycin and incubated with either purified native rat ITPR1 (10 μg/ml) [17], purified human glial fibrillary acidic protein (GFAP) (10 μg/ml) (Merck Chemicals, Darmstadt, Germany) or phosphate buffered saline (PBS) (Euroimmun). The proliferative response was measured by the uptake of ^3^H-thymidine (0.5 μCi/well) added 16 h before harvesting on day 3 of culture. The responses are reported as the mean counts per minute (cpm) values of three cultures.

### Peripheral blood lymphocyte phenotype profiling

Mononuclear blood cells (1.5 × 10^5^) from the patient and the healthy control were cultured in 200 μl culture medium in triplicate together with either ITPR1 protein (10 μg/ml) or GFAP protein (10 μg/ml). Twelve days later, autologous mononuclear blood cells irradiated at 30 Gy were added at a final concentration of 5 × 10^4^ cells per well, as antigen-presenting cells, and the proliferative response was boosted by addition of 10 μg/ml ITPR1 or GFAP protein. The cells were harvested on day 15 of culture. This protocol was used to expand the ITPR1-responding cell lines. No cytokine was added. The proportions of T and B cell subtypes in culture were determined by FACS following the protocol used for peripheral blood lymphocyte phenotype profiling described above.

## Results

### Clinical and electrophysiological findings

*Case 1*. A 60-year-old male heavy smoker (35 pack-years) with no significant neurological history developed severe weakness of the lower legs, which rapidly ascended within 1 week, involving first the thighs and hip muscles, then the muscles of both arms, followed by the respiratory muscles and the mimic muscles on day 6. At first presentation at our department 7 days after onset, severe paresis of the hip flexors (Medical Research Council [MRC] grade 2/5), moderate paresis of the knee flexors (3/5), mild paresis of the feet flexors and extensors (4/5), mild (and also mainly proximal) paresis of the upper limbs (4/5), peripheral facial nerve paresis and mild dysarthrophonia were present. Motor symptoms were accompanied by paraesthesia and hypoaesthesia of the legs, trunk (up to level Th10) and hands and by pallanaesthesia of the upper and lower extremities. Deep tendon reflexes of the lower and upper extremities were absent; Babinski’s sign was negative. Of particular note, most severe pain was present since onset, which ascended from the lower legs to the thighs and to the lumbar region and which required treatment with tilidine, metamizole and gabapentin. Further complaints included impaired micturition and irregular defaecation (with diarrhoea) for several months. Within the last month before onset, the patient had unintentionally lost 5 kg of body weight. MRI of the brain and spinal cord (without gadolinium) showed no abnormalities. Lumbar puncture demonstrated albuminocytologic dissociation and systemic immune activation (Table 1). Nerve conduction studies first revealed severely delayed and missing F waves, indicating damage to the proximal motor nerve axons or anterior horn α-motor neuron cell bodies, A waves, and, later, delayed motor nerve conduction velocities and decreased sensory nerve action potentials (Table 2). Moreover, a marked side difference in motor neuron conduction velocity and distal motor latency of the facial nerve was detected, confirming a right-sided cranial nerve involvement. In addition, involvement of the autonomic nervous system, as indicated by reduced pathological heart rate variability, was noted. Guillain-Barré syndrome (GBS) was suspected, though anti-ganglioside antibodies (GM1-IgG, GM1-IgM, GQ1b-IgG, GQ1b-IgM, GD1a-IgG, GD1a-IgG, GD1b-IgG, GD1b-IgM) were negative. Serology and faecal culture for *Campylobacter* spp., *Salmonella* spp., *Yersinia* spp. and *Shigella* spp. were negative. Serum levels of vitamin B12, B1 and B6, folic acid and vitamin E were normal. To rule out a paraneoplastic aetiology, the patient’s serum was tested for anti-neural antibodies. IHC on brain tissue section revealed high-titre IgG antibodies binding to PCs in a pattern similar to that described for anti-Sj/ITRP1-IgG antibodies [1], and the presence of anti-ITPR1-Ab was subsequently confirmed in two methodologically independent assays, a rat ITPR1-specific dot-blot assay and a human ITRP1-specific CBA (see section “Serological findings” below for details). Treatments with plasma exchange (PEX) (7×) and, subsequently, intravenous immunoglobulins (5 × 25 g) did not result in significant clinical improvement. In line with the lack of treatment response, ITPR1-IgG was still detectable at a titre of 1:1000 (CBA) 7 days after PEX. Chest computed tomography (CT) showed a lesion compatible with lung cancer. Serum neuron-specific enolase, CYFRA21-1 and squamous cell carcinoma antigen levels were normal. A biopsy from the lesion revealed an adenocarcinoma of the lung (TTF1-positive, negative for markers of neuroendocrine differentiation such as chromogranin A and synaptophysin 38). After surgical removal of the tumour (UICC classification: pT1b pN0 [0/18] L0 V0 Pn0 G2 R0), mild clinical improvement was noted, though the patient was still not able to walk or stand. CBA titres had declined to 1:320 by 1 month after operation. Around 1 year after onset, he developed repeat brain infarction, which led to Broca aphasia and brachiofacial hemiparesis on the right side and was attributed to intermittent atrial fibrillation by the then treating physicians. At a follow-up visit, another 4 months later, the paresis of the left arm had completely resolved and only mild paresis of the left leg remained. As sequelae of the two stroke episodes, persisting central facial paresis, complete paresis of the right arm, severe paresis (3/5) of the right leg and motor aphasia were noted. The patient had gained a significant amount of weight (from 48 kg before tumour removal to 60 kg at last follow-up), and regular oncological follow-up examinations had shown no signs of tumour recurrence. Serum anti-Sj/ITPR1-IgG was still detectable, although at lower titre (CBA 1:100).

**Table 1:** Cerebrospinal fluid findings in patient 1

	d6	d24
Total protein	1571 mg/day	225 mg/day
CSF cell count	Normal	Normal
QAlb	34	48
Blood-CSF barrier dysfunction	Yes^a^	Yes^a^
Albuminocytologic dissociation	Yes	Yes
CSF lactate	Normal	Normal
CSF glucose	Normal	Normal
OCBs	Mirror pattern^b^	Mirror pattern^b^
QIgG	Normal	Normal
QIgA	Normal	Normal
QIgM	Normal	Normal
MRZ reaction	Negative	N.d.

No antibodies to *Borrelia burgdorferi, Treponema pallidum* and *Mycobacterium tuberculosis* were detected in CSF and serum. CSF-PCR for EBV, HSV-1, HSV-2 and VZV was negative as well

*QAlb* CSF/serum Alb ratio, *QIgG* CSF/serum IgG ratio, *QIgA* CSF/serum IgA ratio, *QIgM* CSF/serum IgM ratio, *MRZ reaction* measles, rubella, zoster reaction [61–63], *N.d.* not done

^a^As indicated by an elevated CSF/serum albumin ratio

^b^Indicates systemic immune activation

**Table 2:** Electroneuronography and heart rate variability (HRV) findings in patient 1 at days 7 (d7), 24 (d24), 31 (d31), 40 (d40) and 62 (d62), demonstrating axonal and demyelinating sensorimotor poly(radiculo)neuropathy with lost and delayed F waves and autonomic involvement, compatible with Guillain-Barré syndrome

	CMAP [mV]	DML [ms]	mNCV [m/s]	F waves [ms]	A waves	SNAP [μV]	sNCV [m/s]
	Tibial nerve, right					Sural nerve, right	
d7	10.9 (n)	3.8 (n)	41 (n)	*64.3 (↑)*	*Yes*	9.1 (n)	52.6 (n)
d24	6.8 (n)	5.2 (n)	*36.7 (↓)*	*Lost*	*Yes*	6.4 (n)	50 (n)
d40	*2.5 (↓)*	5.5 (n)	*25 (↓)*	*Lost*	N.d.	5.8 (n)	47.5 (n)
d62	*0.6 (↓)*	*8.5 (↑)*	*33 (↓)*	*Lost*	N.d.	N.d.	N.d.
	Ulnar nerve, right					Ulnar nerve, right	
d7	10.6 (n)	2.5 (n)	60 (n)	29.9 (n)	N.d.	*2.3 (↓)*	45.8 (n)
d24	8.6 (n)	2.8 (n)	*38.5 (↓)*	*34.6 (↑)*	N.d.	N.d.	N.d.
d40	9.5 (n)	*3.8 (↑)*	47 (n)	*42.1 (↑)*	N.d.	*Lost*	*Lost*
	Facial nerve, right^a^						
d31		*0.4 (↓* ^*b*^ *)*	*5.2 (↑* ^*b*^ *)*				
	Facial nerve, left^c^						
d31		0.9	4.3				
	HRV [%]						
d7	17 (n)						
d24	*8.7 (↓)*						

Note the continuous deterioration of almost all sensory and motor modalities over the course of the disease. Pathological results are printed in italicized letters

*N.d.* no data, *CMAP* compound muscle action potential, *DML* distal motor latency, *mNCV* motor nerve conduction velocity, *SNAP* sensory nerve action potential, *sNCV* sensory nerve conduction velocity; ↑ increased, ↓ decreased

^a^Symptomatic

^b^Compared with left facial nerve

^c^Asymptomatic

*Case 2*. A 40-year-old man developed bilateral paraesthesia of the lower limbs ascending to the thighs with no manifest paresis. SSEP revealed delayed latency from the lower extremities. Clinical examination revealed hyporeflexia of the lower limbs. The patient's serum IgG bound to murine brain tissue sections in a pattern suggestive of ITPR1-IgG, the presence of which was confirmed by CBA and immunoblot. Bence Jones proteinuria indicated multiple myeloma. In a follow-up sample taken 5 months later, the antibody was still detectable. While the patient’s peripheral symptoms had progressed only slowly, severe depression of unknown cause had occurred in the meantime. Antinuclear and anti-dsDNA antibodies as well as anti-ganglioside antibodies were negative.

*Case 3*. A 79-year-old man developed slowly progressive distal weakness of the upper extremities and disturbance of fine motor skills in the right hand. Some months later, he noted increasing weakness also of the left hand. Upon neurological examination, flaccid paresis (MRC grades 2–4 at first examination, 0–4 at 32 months) and atrophy of the small muscles of both hands, mild paresis of the toe extensors, hypo- and areflexia in the upper and lower extremities, hypoaesthesia of both feet and pallhypoaesthesia in the upper and lower extremities (radius 4/8, patella 2/8, malleus 2/8) were noted. Ganglioside antibodies were repeatedly negative. Electroneuronography demonstrated delayed and lost F waves, A waves, decreased and lost compound muscle action potentials, missing sensory nerve action potential or reduced sensory nerve conduction velocities in the upper and lower extremities and no conduction blocks (Table 3). Electromyography revealed spontaneous activity indicating denervation (fibrillations and positive sharp waves) in five muscles on three levels and fasciculation in six muscles on three levels. The CSF findings (including OCBs) were unremarkable, as was MRI of the cervical spinal cord. Serum ITPR1-IgG was tested for the first time 23 months after onset and was positive at a titre of 1:100 (CBA); both of two follow-up samples were positive as well. No other anti-neural antibodies were detected.

**Table 3:** Electroneuronography findings in patient 3 at 6 months (m6), 9 months (m9) and 32 months (m32) after onset, demonstrating axonal and demyelinating sensorimotor poly(radiculo)neuropathy with, as in patient 1, lost and delayed F waves of the upper and lower extremities

	CMAP [mV]	DML [ms]	mNCV [m/s]	F waves [ms]	A waves	SNAP [μV]	sNCV [m/s]
	Tibial nerve, right					Sural nerve, right	
m6	*1 (↓)*	*7.24 (↑)*	Normal	*67.4 (↑)*	N.d.	N.d.	N.d.
m9	*1.6 (↓)*	Normal	Normal	*56.8 (↑)*	N.d.	N.d.	N.d.
m32	*1.4 (↓)*	Normal	Normal	*Lost*	N.d.	N.d.	N.d.
	Tibial nerve, left					Sural nerve, left	
m6	N.d.	N.d.	N.d.	N.d.	N.d.	*Missing*	*Missing*
m9	*3.1 (↓)*	Normal	Normal	*63.8 (↑)*	N.d.	N.d.	N.d.
m32	*2.6 (↓)*	Normal	Normal	*66 (↑)*	N.d.	*Missing*	*Missing*
	Median nerve, right					Median nerve, right	
m6	*1.4 (↓)*	*5.09 (↑)*	Normal	*Lost*	*Yes*	*7.6 (↓)*	Normal
m32	N.d.	N.d.	N.d.	N.d.	N.d.	Normal	Normal
	Median nerve, left					Median nerve, left	
m6	*2.7 (↓)*	*4.42 (↑)*	Normal	*Lost*	*Yes*	Normal	Normal
	Ulnar nerve, right					Ulnar nerve, right + left	
m6	N.d.	N.d.	N.d.	N.d.	N.d.	N.d.	N.d.
m9	Normal	Normal	Normal	Normal	N.d.	N.d.	N.d.
m32	Normal	Normal	Normal	*33.2 (↑)*	N.d.	N.d.	N.d.
	Ulnar nerve, left					Ulnar nerve, left	
m6	Normal	Normal	Normal	Normal	N.d.	Normal	Normal
m9	N.d.	N.d.	N.d.	N.d.	N.d.	N.d.	N.d.
m32	N.d.	N.d.	N.d.	N.d.	N.d.	Normal	*40 (↓)*
	Peroneal nerve (m. extensor digitorum brevis), left						
m6	*Lost*	*Lost*	*Lost*	*Lost*			
m9	N.d.	N.d.	N.d.	N.d.			
m32	*Lost*	*Lost*	*Lost*	*Lost*			

Pathological results are printed in italicized letters

*N.d.* no data, *CMAP* compound muscle action potential, *DML* distal motor latency, *mNCV* motor nerve conduction velocity, *SNAP* sensory nerve action potential, *sNCV* sensory nerve conduction velocity; ↑ increased; ↓ decreased

### Neuropathological findings

A biopsy sample of the right sural nerve was available from patient 1. The sample was obtained 70 days after onset. Histopathology demonstrated neuritis with several small perivascular cell infiltrates consisting of CD8-positive lymphocytes directly adjacent to small epineural vessels, partly arranged in a bead-like manner and partly invading the adventitia. Analysis of the neurofascicles showed oedema (most pronounced in the subperineural area), a markedly broadened perineurium, severe irregular loss of myelinated axons (with myelin-positive, CD68-positive macrophages present, indicating florid inflammation) and marked endoneural fibrosis. Staining with antibodies to IgG, IgA and IgM revealed severe blood-nerve barrier disruption.

### Serological findings

**Fig. 1:**
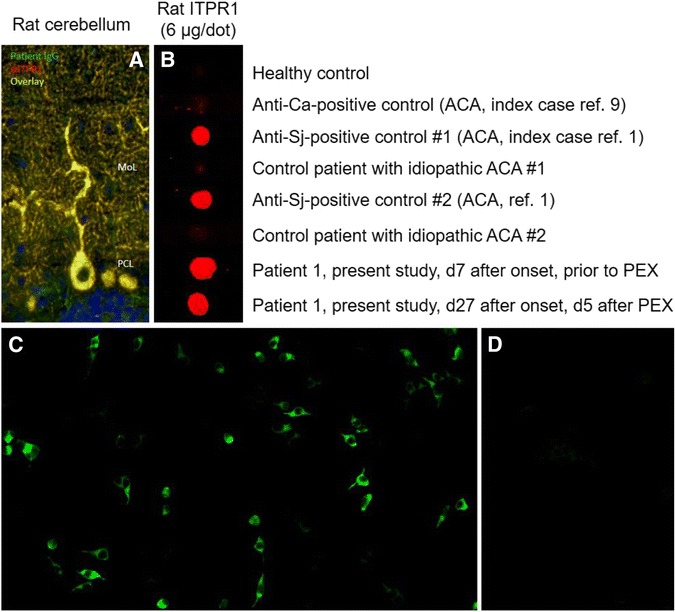
ITPR1-IgG as detected by **a** immunohistochemistry, **b** a dot-blot assay and **c**, **d** a cell-based assay (exemplary findings). **a** Binding of patient serum IgG and of a commercial antibody to ITPR1 to formalin-fixed mouse (as well as rat and primate, not shown) cerebellum tissue sections (*MoL* molecular layer, *PCL* purkinje cell layer). Binding of patient IgG is depicted in *green* (goat anti-human IgG Alexa Fluor® 488); binding of a commercial rabbit anti-ITPR1 antibody in *red* (goat anti-rabbit IgG Alexa Fluor® 568); *yellow* indicates binding of both antibodies. Cell nuclei were stained by 4',6-diamidino-2-phenylindole (*blue colour*). **b** Binding of IgG from the patient’s serum and from two previously reported ITPR1-IgG-positive patients [1] to purified native ITPR1 from rat brain in a dot-blot assay; by contrast, sera from four healthy and disease controls did not bind to ITPR1. A commercial goat anti-human IgG antibody labelled with IRdye®700 (Rockland, Limerick, PA) was used to detect bound patient IgG. **c** Binding of patient IgG to cells transfected with mouse ITPR1 (*left*) but not to mock-transfected HEK293 cells used as control substrate (*right*). Binding of patient IgG was visualized using a goat anti-human IgG secondary antibody labelled with fluorescein isothiocyanate. See the “Methods” section for details. *ACA* autoimmune cerebellar ataxia, *PEX* plasma exchange

Patients 1 and 2 were both positive for ITPR1-IgG as detected by IHC, CBA and immunoblotting. All samples showed the typical staining pattern on cerebellum tissue sections previously described with ITPR1-IgG-positive sera, i.e. staining of the PC somata, dendrites and axons, sparing the PC nucleus as well as all other cells of the cerebellum, at high titres (patient 1: 1:15,000; patient 2: 1:2,500) [1]. Simultaneous incubation of tissue sections with patient IgG and a commercial antibody to ITPR1 resulted in identical staining patterns (Fig. 1); by contrast, no double labelling was seen with healthy control samples. Moreover, both sera reacted with ITPR1-transfected HEK293 cells but not with mock-transfected HEK293 cells used as control substrate in the CBA (patient 1: >1:1,000; patient 2: 1:1,000). Finally, the samples reacted with purified rat ITPR1 in the dot-blot assay (Fig. 1). Patient 3 was not tested at disease onset but only at 23 months, when he was positive for serum ITPR1-IgG as detected by CBA at a titre of 1:100. He remained positive when re-tested 1 month (1:32) and 4 months later (1:10); IHC showed faint staining of PCs at serum dilutions of 1:10 and 1:60.

A broad panel of other anti-neural antibodies (anti-Ca/ARHGAP26, anti-Homer-3, anti-CARPVIII, anti-Hu, anti-Ri, anti-Yo, anti-Tr/DNER, anti-Ma/Ta, anti-GAD65, anti-amphiphysin, anti-CV2/CRMP5, anti-recoverin, anti-SOX1, anti-titin, anti-Zic4, anti-AQP4, anti-MOG, anti-NMDAR, anti-GAGAB receptors, anti-LGI1, anti-CASPR2, anti-glycine receptors, anti-ganglioside antibodies) was negative.

**Fig. 2:**
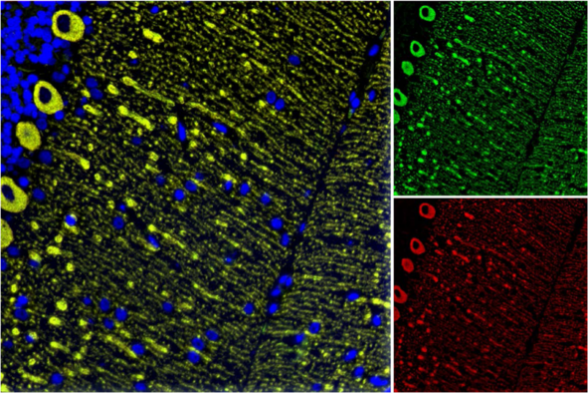
ITPR1-IgG in the CSF. Perfect overlap of the patient IgG from a CSF sample (patient 1) with a commercial antibody to ITPR1 as detected by IHC on a murine cerebellum tissue section. Bound patient IgG is depicted in *green* (Alexa Fluor® 488) and binding of the commercial anti-ITPR1 antibody in *red* (Alexa Fluor® 568); *yellow* indicates areas stained by both antibodies. Cell nuclei were stained by 4',6-diamidino-2-phenylindole (*blue*)

A CSF sample (taken 25 days after onset) was available from patient 1 and was positive for ITPR1 at a titre of 1:32 in the CBA. This corresponded to an ITPR1-IgG AI of 1.65 according to Reiber’s formula (see “Methods” section for details), based on an ITPR1-IgG CBA titre of 1:1000 at the time of lumbar puncture, an IgG CSF/serum ratio of 19.4 and an albumin CSF/serum ratio of 48. When tested at 1:78 dilution, which would correspond to an AI_ITPR1_ of 4, indicating intrathecal synthesis, no ITPR1-IgG antibodies were detectable, suggesting a predominantly extrathecal origin of CSF anti-Sj/ITPR1-IgG in line with the finding of identical (i.e., no CSF-restricted) OCBs in CSF and serum and a normal QIgG in this case. ITPR1-IgG was also detectable in the CSF by IHC on cerebellum tissue sections at a titre of 1:256 (Fig. 2). However, based on a serum titre of 1:15,000, a CSF titre of 1:1182 would have been required to achieve an AI_ITPR1_ of 4, again suggesting an extrathecal synthesis of anti-Sj/ITPR1-IgG in this case. No CSF samples were available for testing from patients 2 and 3.

**Fig. 3:**
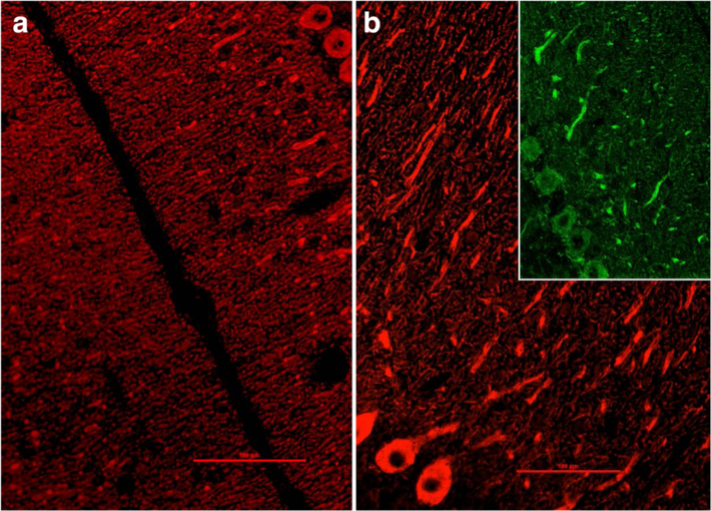
ITPR1 class and subclass analysis revealed IgG1 **a** and IgA (**b**, inset) antibodies in patient 1 and IgG2 antibodies in patient 2 (as well as in patient 3, not shown) **b**

As in the previously reported index case [1], serum ITPR1-IgG belonged exclusively to the IgG1 subclass in the severely affected patient 1 (Fig. 3a). In addition, ITPR1-IgA was present (Fig. 3b, inset) but no ITPR1-IgM. By contrast, serum ITRP1-IgG in the more mildly affected patients 2 and 3 was of the IgG2 subclass (Fig. 3b).

### Immunological findings

#### Demonstration of ITPR1-specific PBMCs by use of a 3H-thymidine proliferation assay

**Fig. 4:**
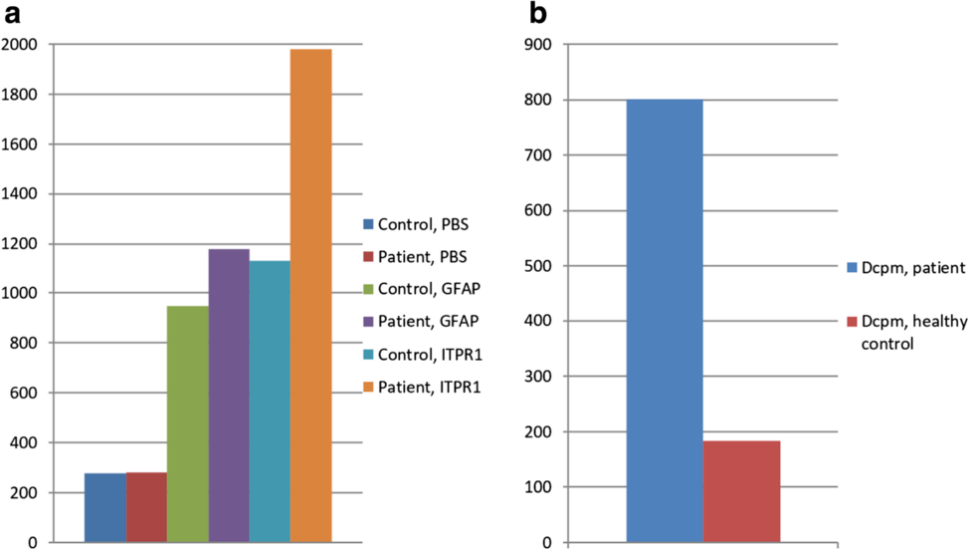
^3^H-thymidine uptake (cpm; mean of three stimulations) in proliferating PBMCs from patient 1 and from a healthy control following stimulation with ITPR1 or GFAP, respectively. **a** Note the significantly higher ^3^H-thymidine uptake in patient PBMCs stimulated with ITPR1 than in the healthy donor; by contrast, no difference was noted after stimulation with GFAP or with PBS. **b** After subtraction of non-antigen-specific, GFAP-induced proliferation, a difference (Δcpm) between patient and healthy control of more than 600 cpm or 340 % was noted following ITPR1 stimulation

After 3 days’ incubation, almost 70 % stronger ^3^H-thymidine uptake was measured in the patient PBMC cultures stimulated with ITPR1 (mean of three cultures 1980 counts per minute (cpm)) than in those stimulated with GFAP (mean 1179 cpm) (Fig. 4a). By contrast, no such strong difference in ^3^H-thymidine uptake was found between ITPR1- (mean 1131 cpm) and GFAP-stimulated (mean 949 cpm) healthy donor control PBMCs. The patient’s ITPR1 response exceeded that of the control patient by 75 % (Fig. 4a). If the control GFAP response was subtracted from the ITPR1 response (Δcpm), the response of the patient’s PBMCs to ITPR1 exceeded that of the healthy control donor PBMCs by 337 % (Δcpm_patient_ 800 cpm vs Δcpm_HC_ 183 cpm) (Fig. 4b). Stimulation with PBS used as negative control resulted in similar ^3^H-thymidine uptake by patient and healthy donor PBMCs (mean of three cultures 280 and 277 cpm, respectively).

#### Demonstration of ITPR1-specific B cells and CD3^+^CD4^+^ T cells among patient PBMCs

**Fig. 5:**
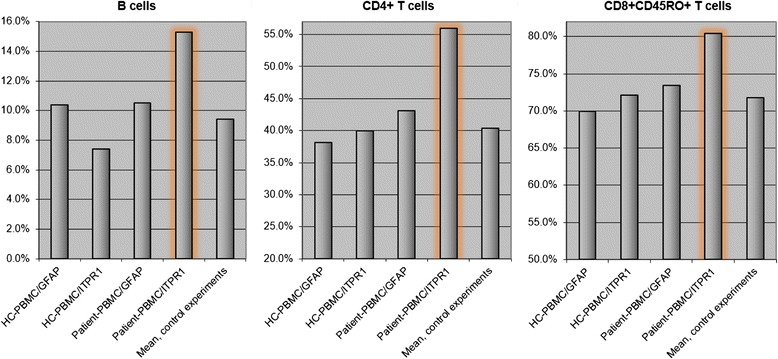
Increase in the proportion of B cells, CD4^+^ T cells and CD8^+^CD45RO^+^ T cells among PBMCs from patient 1 (2 × 10^5^ cells) following stimulation with ITPR1 (10 μg/ml) (*orange outlines*) compared with stimulation with GFAP (10 μg/ml) and compared with healthy donor PBMCs stimulated with the same amount of ITPR1 or GFAP, respectively

The proportion of B cells among total patient PBMCs was higher after ITPR1 stimulation (15.3 %) than after GFAP stimulation (10.5 %). This corresponded to an absolute difference of 45 %. By contrast, ITPR1 was even a slightly weaker stimulus than GFAP when used to stimulate healthy control PBMCs (Fig. 5). The proportion of CD3^+^CD4^+^ cells among total patient PBMCs was also higher after ITPR1 stimulation (56 %) than after GFAP stimulation (43.1 %). This corresponded to an absolute difference of 30 %. By contrast, the difference was only 1.9 % when healthy control samples were used (Fig. 5). While no such difference between ITPR1 and GFAP stimulation was found for total CD8 cells (neither among patient nor among healthy control PBMCs) (not shown), an around 10 % higher proportion of memory cells (CD3^+^CD8^+^CD45RO^+^ phenotype) among total CD8 T cells was found after 15-day stimulation with ITPR1 than after stimulation for the same period of time with GFAP (Fig. 5). No relevant differences were found for the other markers investigated (see *Methods *section for details).

#### Demonstration of ITPR1 expression in the spinal cord and peripheral nervous system

**Fig. 6:**
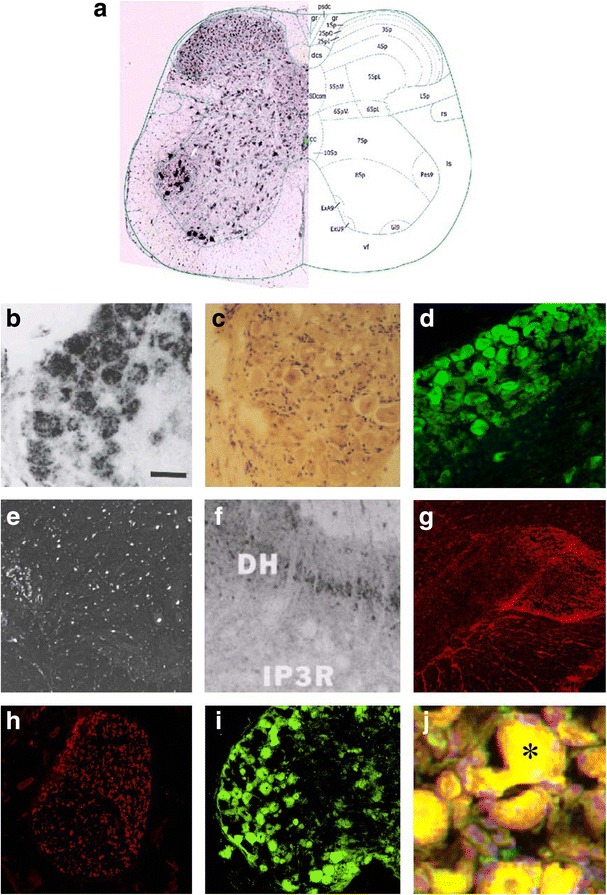
Expression of ITPR1 in the spinal cord (**a**, **e**–**g**), sural nerve (**h**) and dorsal root ganglia (DRG) (**b**–**d**, **i**–**j**) of adult mouse (**a**, **d**), rat (**b**–**c**, **e**–**f**, **i**–**j**) and rhesus monkey (**g**–**h**) and as detected by in situ hybridization (**a**, **b**, **e**) and immunostaining (**c**–**d**, **f**–**j**), and binding of IgG from the patient’s CSF (**i**) and serum (**j**) to sensory neuron somata in rat DRG as detected by indirect immunofluorescence. A polyclonal rabbit antibody to ITPR1 (Dianova) and an AlexaFluor®568-labelled goat anti-rabbit IgG secondary antibody were used to detect ITPR1 expression (*red*). A fluorescein isothiocyanate-labelled goat anti-human IgG antibody was used to visualize bound patient IgG (*green*). *Yellow areas* in **j** indicate overlay of the patient’s IgG and the commercial ITPR1 antibody. **a** Taken from the Allen Spinal Cord Atlas [64] (http://mousespinal.brain-map.org/). **b**, **c** and **e** Reproduced from Dent et al. [2] with kind permission of the publisher , **d** from Zhuang et al. [5] (made available under the Creative Commons CC0 public domain dedication; https://creativecommons.org/publicdomain/zero/1.0/) , and **f** from Sharp et al. [3] with kind permission of the publisher. *DH* dorsal horn, *IP3R* inositol-1,4,5 trisphosphate receptor

ITPR1 has been previously shown to be expressed also in the posterior and anterior horn of the spinal cord [2, 3] and in the sensory neuron somata in the DRG (Fig. 6). We were able to reproduce those previous findings using a commercial antibody to ITPR1 (Fig. 6g, j). In addition, we demonstrated expression of ITPR1 by the sural nerve (Fig. 6h). To exclude non-specific binding or cross-reactivity of the commercial antibody used, we employed a second commercial antibody to ITPR1 (Alomone Labs, Jerusalem, Israel) resulting in an identical binding pattern (not shown). As a proof of principle, we incubated serum samples from patient 1 and from two further ITPR1-IgG-positive patients with rat DRG tissue sections and found strong binding to the sensory DRG neurons in a pattern identical to that seen with the commercial ITPR1 antibody (Fig. 6i, j).

#### Demonstration of ITPR1 expression by the patient’s adenocarcinoma

**Fig. 7:**
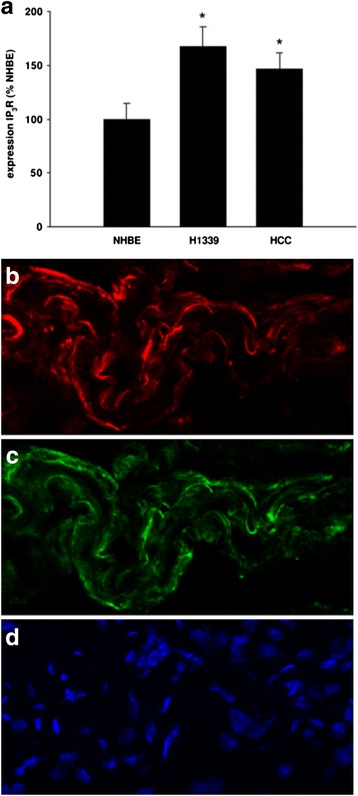
ITPR1 is expressed in a subset of carcinomas of the lung. **a** Quantitative Western blot analysis showing ITPR1 expression as percentage of the ITPR1 expression in normal human bronchial epithelial (NHBE) cells, in the small cell lung cancer cell line H1339 and in the non-small cell lung cancer cell line HCC. The expression of IP3R was increased with H1339 showing the highest expression (*n* = 4, *P < 0.01 versus all other groups). Image reproduced from Bergner et al. (2009) [19] (distributed under the terms of the Creative Commons Attribution License [http://creativecommons.org/licenses/by/2.0]). **b**–**d** Indirect immunofluorescence-based immunohistochemistry assay showing ITPR1 expression within adenocarcinoma tissue obtained from patient 1 (**b** binding of a commercial rabbit antibody to ITPR1 visualized using a goat anti-rabbit IgG Alexa Fluor® 568-labelled secondary antibody [*red fluorescence*]; **c** binding of patient serum IgG visualized by use of a goat anti-human IgG secondary antibody labelled with Alexa Fluor® 488 [*green fluorescence*]; **d** staining of cell nuclei by DAPI [*blue fluorescence*])

ITPR1 has been shown to be expressed in a subset of adenocarcinomas of the lung as demonstrated by Western blot and conventional immunohistochemistry; by contrast, no or only low levels of ITPR1 protein expression have been reported in normal lung tissue [18, 19]. Incubation of the only cryosections of the adenocarcinoma of patient 1 available for research with a commercial antibody to ITPR1 revealed ITPR1 expression by a subset of cells which was identical to that obtained by incubating the sections with an ITPR1-IgG-positive CSF sample from the same patient (Fig. 7).

## Discussion

Autoantibodies to ITPR1 (also termed anti-Sj after the index sample) were originally described by some of us in patients with ACA [1, 20–22]. The results from the present study suggest that the spectrum of symptoms associated with autoimmunity to ITPR1 may be broader than initially thought and may include motor, sensory (including severe pain) and autonomic symptoms compatible with (myelo)radiculopathy and/or peripheral neuropathy.

### Clinical and aetiopathogenetic considerations

The latter hypothesis would be well in line with the fact that ITPR1 is not exclusively expressed in the brain but is present also in the anterior and posterior (including the substantia gelatinosa) horns of the spinal cord [2, 3], in the sensory DRG, in the trigeminal ganglia, in the sympathetic ganglia [2], and in the perip﻿heral nerves. Accordingly, signs and symptoms of peripheral nervous system (PNS) involvement (reduced sensory conduction velocity of sural nerve and motor conduction velocities of the median and tibial nerves; reduced muscle stretch reflexes and vibration sense) have been described in a subset of patients with mutations in the *ITRP1* gene [23, 24], and dysregulation of ITPR1 has been found to cause allodynia and hyperalgesia in an animal model [5].

The presence of lung cancer suggests a paraneoplastic aetiology in patient 1. Lung cancer is the most common type of tumour in patients with paraneoplastic neurological disorders (PND). However, no antibodies typically found in patients with lung cancer and PND [25], including anti-Hu, anti-CV2/CMRP5, anti-amphiphysin, anti-VGCC, anti-SOX1, anti-GABABR and anti-AMPAR, were present in our patients. ITPR1 has been shown to be expressed in a broad variety of tumours (partly ectopically), particularly in Hodgkin disease (HD) and malignant lymphoid and myeloid cell lines, but also lung cancer, melanoma, prostate cancer and many other tumour entities [18]. In patient 2, Bence Jones proteinuria in fact indicated the presence of a non-Hodgkin (B cell) lymphoma and thus a possible paraneoplastic aetiology in this patient as well. By contrast, no evidence of cancer was present in patient 3 at last follow-up.

Patient 1 presented with ascending motor and sensory symptoms suggestive of GBS. Subacute sensory neuronopathy (Denny-Brown syndrome) and subacute motor neuropathy both represent “classical paraneoplastic syndromes” according to an international consensus definition [25]; GBS, acute sensorimotor neuropathy and autonomic neuropathy, on the other hand, are well-recognized “non-classical”, non-obligatory paraneoplastic syndromes [25]. While GBS is considered to be of non-paraneoplastic origin in the vast majority of cases, numerous reports of GBS in oncological patients exist, and a population-based study found an increased frequency for tumours in patients with GBS (odds ratio ~2.4) [26]. Associated tumours reported in GBS were mainly lung cancer, Hodgkin lymphoma, non-Hodgkin lymphomas and leukaemia, though several other types of cancer were occasionally described (see Additional file 1: Table S1 for a comprehensive list and references).

A paraneoplastic aetiology of ITPR1-IgG-associated autoimmunity in patient 1 is further suggested by the finding of ITRP1 expression in the tumour tissue in structures also stained by the patient’s IgG. According to proteomic data, ITPR1 is not constitutively expressed in normal lung tissue or only at low level [18]. Of note, ITPR1 has been detected at protein level only in a subset of adenocarcinomas of the lung [18, 19], leaving the possibility that anti-ITPR1 seropositivity in patients may denote a subtype of adenocarcinoma in patients with lung cancer and PND.

Whether anti-ITPR1 autoimmunity has a protective role in patients with PND, as suggested for other antibody-related syndromes such as anti-Hu, is currently unknown but warrants further investigation. Of potential interest in this context, ITPR1 has been reported to protect renal cancer cells from NK-mediated lysis [27]. In vivo, ITPR1 targeting significantly enhanced NK-mediated tumour regression [27].

Some paraneoplastic disorders, including anti-Hu, anti-CV2/CRMP5 and anti-amphiphysin syndrome, have been shown to be capable of causing both central and peripheral neurological symptoms, in line with the presence of the respective autoantigens in both central and peripheral nervous tissue. Similarly, ITPR1 is expressed both in the CNS and in the PNS. Why one and the same paraneoplastic neurological disorder affects the CNS in some patients but the PNS in others (and both systems in yet others) is not well understood. However, differences in blood-brain/CSF barrier function among patients or variations hereof over time in the same patient, the presence or absence of antigen-specific intrathecal B and T cell clones, differences in antigen/epitope availability among tissues and, importantly, differences in antibody epitope/isoform specificity could play a role. To date, eight isoforms of ITPR1 produced by alternative splicing have been described [28]. Future studies need to address (a) possible cross-reactivity of anti-ITPR1-IgG with ITPR2 and ITPR3 as well as among the various ITPR1 isoforms and (b) differences in epitope/isoform specificity between patients with ITPR1-IgG-associated ACA and ITPR1-IgG-positive patients with peripheral nervous system involvement.

In the adult brain, ITPR1 is not only expressed in PCs of the cerebellum but also in hippocampal neurons (particularly in CA1 pyramidal cells) [2–4], in the putamen and the caudate nucleus, and in the cerebral cortex (most pronounced in pyramidal layer V and non-pyramidal layer II) [2, 3]. Given this wide expression profile, it is likely that the spectrum of ITPR1 autoimmunity will broaden further as more patients are identified in the future. In this context, it is worth noting that patient 2 developed severe depression in the course of disease. Signs of possible limbic involvement (confusion, severe memory loss, suspected temporal lobe epilepsy) were also present in two recent ITPR1-IgG-positive patients identified by the authors, one of whom also had cerebellar ataxia (unpublished data).

In the light of ITPR1 being expressed in the autonomic nervous system, in particular in the sympathetic ganglia [2], it is of special note that patient 1 exhibited reduced pathological heart frequency variability, a typical sign of autonomic neuropathy of the heart, and later developed repeated stroke episodes attributed to intermittent atrial fibrillation. Interestingly, ITPR1 is also present in human and murine cardiac myocytes [18, 29, 30], where it is involved in crucial regulation of intracellular calcium pathways: The cardiac ITPR receptors have recently been shown to modulate the electromechanical properties of the human myocardium and its propensity to develop arrhythmias [29]. Further studies on anti-ITPR1 autoimmunity should therefore pay attention also to signs and symptoms of autonomic heart dysregulation and autoimmune myocarditis.

It is of further interest in this context that patient 1 reported continuous bowel dysfunction with diarrhoea starting several months before onset of the polyradiculoneuropathy. Whether these symptoms were caused by infection and whether an infectious agent was involved in the pathogenesis of the patient’s neurological disorder (e.g. molecular mimicry) remains unknown. No evidence was found for an infection with *Campylobacter jejuni*, which is present in around 30 % of patients with classical GBS. Alternatively, the patient’s bowel dysfunction could have been a first sign of autonomic neuropathy. The latter hypothesis is supported by the fact that he simultaneously developed bladder dysfunction. ITPR1 is expressed in the sympathetic ganglia as well as in the smooth muscle cells of the bowel and the bladder. Accordingly, we found binding of IgG from this and other ITPR1-IgG-positive patients to smooth muscle cells in the enteric wall in a pattern identical to that seen with a commercial antibody to ITPR1 [1]. Moreover, paraneoplastic enteropathy is a well-known complication of cancer, and diarrhoea has recently been recognized as a typical prodromal symptom in DPPX (dipeptidyl-aminopeptidase-like protein 6) syndrome, another novel antibody-related autoimmune disease of the CNS [31–34]. Studies evaluating the frequency of ITPR1-Ab in patients with paraneoplastic and other types of suspected autonomic enteropathy seem therefore warranted.

Finally, the facts that ITPR1 is expressed in the sensory DRG, the trigeminal ganglia and the substantia gelatinosa [3], in which C fibre axons synapse with neurons of the pain-transmitting lateral spinothalamic tract and damage to which can cause pain and hyperalgesia, and that ITPR1 dysregulation has been implicated in hyperalgesia and allodynia in animal studies [5], along with the presence of a severe pain syndrome in our patient, provide a preliminary rationale for seeking a potential role of ITPR1-related autoimmunity also in patients with pain syndromes of unknown aetiology.

### Immunological considerations

Passive transfer experiments using IgG from anti-ITPR1-positive patients have not yet been performed. Therefore, no direct evidence for a pathogenic impact of the antibody is currently available. ITPR1 is thought to be primarily an intracellular antigen located in membranes encompassing the endoplasmatic and, in muscle cells, sarcoplasmatic reticulum. Many researchers believe that intracellular antigens are not accessible to antibodies in vivo. In fact, most neurological autoantibodies of proven pathogenic impact, such as antibodies to AQP4 in neuromyelitis optica [35–38], acetylcholine receptor in myasthenia gravis, VGCC in Lambert-Eaton syndrome [39] and mGluR1 in paraneoplastic cerebellar degeneration [40], target cell-surface-expressed proteins. Moreover, passive transfer of antibodies to intracellular antigens such as anti-Yo [41–43] has not produced clinical disease in animal studies. Instead, T cell-mediated immune mechanisms directed against the target antigen of the accompanying antibody have been proposed to play a role in those disorders [44–47]. It is therefore possible that the antibody has diagnostic but no pathogenic impact, similar to the situation in many paraneoplastic neurological disorders.

However, surface localization of ITPR1 has been reported to occur under certain circumstances [48–52], warranting further investigation. Moreover, studies demonstrating cell damage following uptake of paraneoplastic antibodies by neurons have somewhat challenged the above-mentioned paradigm (see [20–22] for a summary). Indirect evidence suggesting a potential pathogenic role of anti-ITPR1 includes the fact that the antibodies mainly belonged to the complement-activating IgG1 subclass, the very high serum titres in two of our patients (titres were lower in patient 2, in whom ITPR1 was tested late in the disease course), the decline in titres after tumour removal, which was accompanied by clinical stabilization and improvement, and the good concordance of the antigen’s tissue expression profile with the clinical symptoms present in our patient, in particular in patient 1. The absence of intrathecal ITPR1-IgG synthesis does not *per se* argue against a pathogenic role of the antibody: First, the antibody can still reach the CSF via passive diffusion and/or a leaky blood-brain/CSF barrier (ITPR1 was in fact present in the CSF and Q_Alb_ markedly elevated in patient 1). Second, the blood-CSF barrier is thought to be particularly leaky around the nerve roots. Third, there is no need for intrathecal synthesis when it comes to peripheral nerve damage. Finally, AIs are regularly negative also in AQP4-IgG-positive neuromyelitis optica spectrum disorders (NMOSD) [53] and in MOG-IgG-positive encephalomyelitis [54–58], two diseases in which a direct pathogenic impact of the antibody has been proven or is﻿ highly likely.

Of note, anti-Sj/﻿ITPR1-IgG was of the strongly complement-activating IgG1 subclass in the severely affected patient 1 (as well as in the severely disabled anti-﻿Sj/ITPR1-IgG index patient [1]), but exclusively of the very weakly complement-activating IgG2 subclass in patient 2, who had relatively mild disease with no major progression of his polyneuropathy after 1.5 years. Future studies investigating the clinical associations of ITPR1-related autoimmunity should therefore include IgG subclass analyses.

To investigate the role of cell-mediated immunity in this disease, we studied the proliferative response of the PBMCs from patient 1 and from a control donor to stimulation with purified ITPR1 (boosted with radiated antigen-presenting cells) and a control protein in vitro, and assessed the PBMC surface phenotypes before and after stimulation. Phenotypic analysis of the PBMCs revealed a marked difference in proportions of B cells, CD4 T cells and CD8 memory T cells between patient and control donor after exposure to ITPR1. Moreover, compared with the healthy donor the patient’s PBMCs proliferated strongly as measured by ^3^H-thymidine uptake after ITPR1 stimulation. Taken together, these findings indicate that the patient’s PBMCs contained ITPR1-specific B cells and autoreactive CD4 and CD8 T cells, which could be directly involved in injury of the nervous system as well as in antitumour immunity.

### Serological and therapeutic considerations

Anti-Sj/ITPR1-IgG titres were extraordinarily high both in the IHC assay (up to 1:15,000) and in the CBA (up to >1:1000) during acute disease in patient 1; after removal of the tumour (and thus of the ectopically presented antigen), CBA titres declined to 1:320 and later 1:100. Similarly, relatively high titres of anti-Sj/ITPR1-IgG were noted in patient 2 (IHC 1:5,000) and in all of the previously reported ITPR1-IgG-positive cases (IHC 1:5,000, 1:3,200, 1:3,200, 1:1,000) [1]. In direct comparison, IHC titres were higher on average than those in the majority of patients with AQP4-IgG-positive NMOSD or MOG-IgG-positive encephalomyelitis tested by the authors in recent years using the same methodology [59, 60].

Patients 1 and 2 yielded a positive result in all three assays employed, largely ruling out false-positive results due to insufficient assay specificity. While mouse ITPR1 was used in the CBA, purified rat was employed in the dot-blot assay, and primate, rat and mouse ITPR1 was the antigenic substrate in the IHC assay. Similarly, all previously reported patients reacted with all three antigens. This suggests that the epitope targeted by ITPR1-IgG in these patients was located in a region not relevantly affected by differences in amino acid sequence and/or confirmation between those species. Patient 3 was positive in the CBA and IHC assay but negative in the dot-blot assay; while the reason remains unknown, it is likely that this was due to sensitivity issues related to the dot-blot assay, given that the sample repeatedly yielded low titres in the CBA and in the IHC assay.

Interestingly, serum anti-Sj/ITPR1-IgG was still detected at a titre of >1:1000 both in the IHC assay and in the CBA after seven PEX treatments in patient 1, and CSF ITRP1 was positive 18 days after the first PEX treatment in that patient. This is in line with the authors’ experience from other autoantibody-related neurological syndromes, in which the pathogenic antibodies occasionally persist after five to seven PEX treatments, indicating that the number of PEX treatments usually administered in neurological indications may be too low to achieve complete autoantibody removal in some cases and may need to be adjusted according to serological findings. As a caveat, however, it should be kept in mind that evidence for a direct pathogenic role of the antibody is lacking so far and that the immune response in patients with paraneoplastic neurological syndromes may contribute to controlling tumour growth and spread. Moreover, T cells may be involved in the pathogenesis, which would make it necessary to employ treatment modalities targeting not only the B cell and antibody arm of the immune reaction but also the T cell arm.

## Conclusions

In conclusion, our study expands the spectrum of clinical manifestations associated with anti-Sj/ITPR1-IgG and suggests that autoimmunity to ITPR1 may possibly be involved in the pathogenesis of peripheral neuropathy in selected patients, including in cases clinically suggestive of GBS. Moreover, the presence of malignant tumours in two of our patients as well as the expression of ITPR1 within the tumour in one of them suggests a paraneoplastic aetiology of ITPR1 at least in a subset of patients. Studies on the frequency and syndrome specificity of anti-Sj/ITPR1-IgG in patients with PNS disorders, including, among others, GBS, autonomic neuropathies and pain syndromes of unknown aetiology, are now warranted. Lung cancer, haematological tumours and a paraneoplastic origin should be considered in anti-Sj/ITPR1-IgG-positive patients.

## Additional file

Additional file 1: Table S1. List of tumours previously reported in association with Guillain-Barré syndrome. (PDF 94 kb)

## Abbreviations

Ab: Antibody
ACA: Autoimmune cerebellar ataxia
AI: Antibody index
AMPAR: A-amino-3-hydroxy-5-methyl-4-isoxazolepropionic acid receptor
ARHGAP26: RhoGTPase-activating protein 26
BSA: Bovine serum albumin
CBA: Cell-based assay
CHAPS: 3-[(3-cholamidopropyl)dimethylammonio]-1-propanesulfonate
CSF: Cerebrospinal fluid
CT: Computed tomography
DPPX: Dipeptidyl-aminopeptidase-like protein 6
DRG: Dorsal root ganglia
FACS: Fluorescence-activated cell sorting
FITC: Fluorescein isothiocyanate
GABABR: Gamma-aminobutyric acid B receptors
GBS: Guillain-Barré syndrome
GFAP: Glial fibrillary acidic protein
HEK: Human embryonic kidney cells
IgG: Immunoglobulin G
IHC: Immunohistochemistry
ITPR1: Inositol 1,4,5-trisphosphate type 1 receptor
MoL: Molecular layer
MRC: Medical Research Council s﻿cale
NMOSD: Neuromyelitis optica spectrum disorders
PBMC: Peripheral blood mononuclear cell
PBS: Phosphate buffered saline
PC: Purkinje cells
PCL: PC layer
PEX: Plasma exchange
PND: Paraneoplastic neurological disorder
PNS: Peripheral nervous system
RT: Room temperature
TBS: Tris-buffered saline
VGCC: Voltage-gated calcium channels

## References

[1] Jarius S, Scharf M, Begemann N, Stocker W, Probst C, Serysheva II, Nagel S, Graus F, Psimaras D, Wildemann B, Komorowski L (2014). Antibodies to the inositol 1,4,5-trisphosphate receptor type 1 (ITPR1) in cerebellar ataxia. J Neuroinflammation.

[2] Dent MA, Raisman G, Lai FA (1996). Expression of type 1 inositol 1,4,5-trisphosphate receptor during axogenesis and synaptic contact in the central and peripheral nervous system of developing rat. Development.

[3] Sharp AH, Dawson TM, Ross CA, Fotuhi M, Mourey RJ, Snyder SH (1993). Inositol 1,4,5-trisphosphate receptors: immunohistochemical localization to discrete areas of rat central nervous system. Neuroscience.

[4] Nakanishi S, Maeda N, Mikoshiba K (1991). Immunohistochemical localization of an inositol 1,4,5-trisphosphate receptor, P400, in neural tissue: studies in developing and adult mouse brain. J Neurosci.

[5] Zhuang GZ, Keeler B, Grant J, Bianchi L, Fu ES, Zhang YP, Erasso DM, Cui JG, Wiltshire T, Li Q (2015). Carbonic anhydrase-8 regulates inflammatory pain by inhibiting the ITPR1-cytosolic free calcium pathway. PLoS One.

[6] Ludtke SJ, Tran TP, Ngo QT, Moiseenkova-Bell VY, Chiu W, Serysheva II (2011). Flexible architecture of IP3R1 by Cryo-EM. Structure.

[7] Doss S, Nümann A, Ziegler A, Siebert E, Borowski K, Stöcker W, Prüss H, Wildemann B, Endres M, Jarius S (2014). Anti-Ca/anti-ARHGAP26 antibodies associated with cerebellar atrophy and cognitive decline. J Neuroimmunol.

[8] Jarius S, Martinez-Garcia P, Hernandez AL, Brase JC, Borowski K, Regula JU, Meinck HM, Stocker W, Wildemann B, Wandinger KP (2013). Two new cases of anti-Ca (anti-ARHGAP26/GRAF) autoantibody-associated cerebellar ataxia. J Neuroinflammation.

[9] Jarius S, Wandinger KP, Horn S, Heuer H, Wildemann B (2010). A new Purkinje cell antibody (anti-Ca) associated with subacute cerebellar ataxia: immunological characterization. J Neuroinflammation.

[10] Reiber H, Lange P (1991). Quantification of virus-specific antibodies in cerebrospinal fluid and serum: sensitive and specific detection of antibody synthesis in brain. Clin Chem.

[11] Jarius S, Eichhorn P, Wildemann B, Wick M (2012). Usefulness of antibody index assessment in cerebrospinal fluid from patients negative for total-IgG oligoclonal bands. Fluids Barriers CNS.

[12] Stich O, Jarius S, Kleer B, Rasiah C, Voltz R, Rauer S (2007). Specific antibody index in cerebrospinal fluid from patients with central and peripheral paraneoplastic neurological syndromes. J Neuroimmunol.

[13] Reiber H (1998). Cerebrospinal fluid—physiology, analysis and interpretation of protein patterns for diagnosis of neurological diseases. Mult Scler.

[14] Reiber H, Ungefehr S, Jacobi C (1998). The intrathecal, polyspecific and oligoclonal immune response in multiple sclerosis. Mult Scler.

[15] Reiber H (1994). Flow rate of cerebrospinal fluid (CSF)—a concept common to normal blood-CSF barrier function and to dysfunction in neurological diseases. J Neurol Sci.

[16] Haas J, Bekeredjian-Ding I, Milkova M, Balint B, Schwarz A, Korporal M, Jarius S, Fritz B, Lorenz HM, Wildemann B (2011). B cells undergo unique compartmentalized redistribution in multiple sclerosis. J Autoimmun.

[17] Serysheva II, Bare DJ, Ludtke SJ, Kettlun CS, Chiu W, Mignery GA (2003). Structure of the type 1 inositol 1,4,5-trisphosphate receptor revealed by electron cryomicroscopy. J Biol Chem.

[18] Uhlen M, Fagerberg L, Hallstrom BM, Lindskog C, Oksvold P, Mardinoglu A, Sivertsson A, Kampf C, Sjostedt E, Asplund A (2015). Proteomics. Tissue-based map of the human proteome. Science.

[19] Bergner A, Kellner J, Tufman A, Huber RM (2009). Endoplasmic reticulum Ca2 + −homeostasis is altered in small and non-small cell lung cancer cell lines. J Exp Clin Cancer Res.

[20] Jarius S, Wildemann B (2015). 'Medusa-head ataxia': the expanding spectrum of Purkinje cell antibodies in autoimmune cerebellar ataxia. Part 1: Anti-mGluR1, anti-Homer-3, anti-Sj/ITPR1 and anti-CARP VIII. J Neuroinflammation.

[21] Jarius S, Wildemann B (2015). 'Medusa head ataxia': the expanding spectrum of Purkinje cell antibodies in autoimmune cerebellar ataxia. Part 2: Anti-PKC-gamma, anti-GluR-delta2, anti-Ca/ARHGAP26 and anti-VGCC. J Neuroinflammation.

[22] Jarius S, Wildemann B (2015). 'Medusa head ataxia': the expanding spectrum of Purkinje cell antibodies in autoimmune cerebellar ataxia. Part 3: Anti-Yo/CDR2, anti-Nb/AP3B2, PCA-2, anti-Tr/DNER, other antibodies, diagnostic pitfalls, summary and outlook. J Neuroinflammation.

[23] Hara K, Shiga A, Nozaki H, Mitsui J, Takahashi Y, Ishiguro H, Yomono H, Kurisaki H, Goto J, Ikeuchi T (2008). Total deletion and a missense mutation of ITPR1 in Japanese SCA15 families. Neurology.

[24] Hara K, Fukushima T, Suzuki T, Shimohata T, Oyake M, Ishiguro H, Hirota K, Miyashita A, Kuwano R, Kurisaki H (2004). Japanese SCA families with an unusual phenotype linked to a locus overlapping with SCA15 locus. Neurology.

[25] Darnell RB, Posner JB (2011). Paraneoplastic syndromes.

[26] Vigliani MC, Magistrello M, Polo P, Mutani R, Chio A (2004). Risk of cancer in patients with Guillain-Barre syndrome (GBS). A population-based study. J Neurol.

[27] Messai Y, Noman MZ, Hasmim M, Janji B, Tittarelli A, Boutet M, Baud V, Viry E, Billot K, Nanbakhsh A (2014). ITPR1 protects renal cancer cells against natural killer cells by inducing autophagy. Cancer Res.

[28] Nucifora FC, Li SH, Danoff S, Ullrich A, Ross CA (1995). Molecular cloning of a cDNA for the human inositol 1,4,5-trisphosphate receptor type 1, and the identification of a third alternatively spliced variant. Brain Res Mol Brain Res.

[29] Signore S, Sorrentino A, Ferreira-Martins J, Kannappan R, Shafaie M, Del Ben F, Isobe K, Arranto C, Wybieralska E, Webster A (2013). Inositol 1, 4, 5-trisphosphate receptors and human left ventricular myocytes. Circulation.

[30] Przyklenk K, Maynard M, Whittaker P (2006). First molecular evidence that inositol trisphosphate signaling contributes to infarct size reduction with preconditioning. Am J Physiol Heart Circ Physiol.

[31] Balint B, Jarius S, Nagel S, Haberkorn U, Probst C, Blocker IM, Bahtz R, Komorowski L, Stocker W, Kastrup A (2014). Progressive encephalomyelitis with rigidity and myoclonus: a new variant with DPPX antibodies. Neurology.

[32] Piepgras J, Holtje M, Michel K, Li Q, Otto C, Drenckhahn C, Probst C, Schemann M, Jarius S, Stocker W (2015). Anti-DPPX encephalitis: pathogenic effects of antibodies on gut and brain neurons. Neurology.

[33] Stoeck K, Carstens PO, Jarius S, Raddatz D, Stocker W, Wildemann B, Schmidt J (2015). Prednisolone and azathioprine are effective in DPPX antibody-positive autoimmune encephalitis. Neurol Neuroimmunol Neuroinflamm.

[34] Boronat A, Gelfand JM, Gresa-Arribas N, Jeong H-Y, Walsh M, Roberts K, Martinez-Hernandez E, Rosenfeld MR, Balice-Gordon R, Graus F (2012). Encephalitis and antibodies to DPPX, a subunit of Kv4.2 potassium channels. Ann Neurol.

[35] Jarius S, Wildemann B, Paul F (2014). Neuromyelitis optica: clinical features, immunopathogenesis and treatment. Clin Exp Immunol.

[36] Levy M, Wildemann B, Jarius S, Orellano B, Sasidharan S, Weber MS, Stuve O (2014). Immunopathogenesis of neuromyelitis optica. Adv Immunol.

[37] Jarius S, Paul F, Franciotta D, Waters P, Zipp F, Hohlfeld R, Vincent A, Wildemann B (2008). Mechanisms of disease: aquaporin-4 antibodies in neuromyelitis optica. Nat Clin Pract Neurol.

[38] Jarius S, Wildemann B (2010). AQP4 antibodies in neuromyelitis optica: diagnostic and pathogenetic relevance. Nat Rev Neurol.

[39] Vincent A, Lang B, Newsom-Davis J (1989). Autoimmunity to the voltage-gated calcium channel underlies the Lambert-Eaton myasthenic syndrome, a paraneoplastic disorder. Trends Neurosci.

[40] Sillevis Smitt P, Kinoshita A, De Leeuw B, Moll W, Coesmans M, Jaarsma D, Henzen-Logmans S, Vecht C, De Zeeuw C, Sekiyama N (2000). Paraneoplastic cerebellar ataxia due to autoantibodies against a glutamate receptor. N Engl J Med.

[41] Graus F, Illa I, Agusti M, Ribalta T, Cruz-Sanchez F, Juarez C (1991). Effect of intraventricular injection of an anti-Purkinje cell antibody (anti-Yo) in a guinea pig model. J Neurol Sci.

[42] Tanaka K, Tanaka M, Igarashi S, Onodera O, Miyatake T, Tsuji S (1995). Trial to establish an animal model of paraneoplastic cerebellar degeneration with anti-Yo antibody. 2. Passive transfer of murine mononuclear cells activated with recombinant Yo protein to paraneoplastic cerebellar degeneration lymphocytes in severe combined immunodeficiency mice. Clin Neurol Neurosurg.

[43] Tanaka K, Tanaka M, Onodera O, Igarashi S, Miyatake T, Tsuji S (1994). Passive transfer and active immunization with the recombinant leucine-zipper (Yo) protein as an attempt to establish an animal model of paraneoplastic cerebellar degeneration. J Neurol Sci.

[44] Tanaka M, Tanaka K, Tsuji S, Kawata A, Kojima S, Kurokawa T, Kira J, Takiguchi M (2001). Cytotoxic T cell activity against the peptide, AYRARALEL, from Yo protein of patients with the HLA A24 or B27 supertype and paraneoplastic cerebellar degeneration. J Neurol Sci.

[45] Benyahia B, Liblau R, Merle-Beral H, Tourani JM, Dalmau J, Delattre JY (1999). Cell-mediated autoimmunity in paraneoplastic neurological syndromes with anti-Hu antibodies. Ann Neurol.

[46] Rousseau A, Benyahia B, Dalmau J, Connan F, Guillet JG, Delattre JY, Choppin J (2005). T cell response to Hu-D peptides in patients with anti-Hu syndrome. J Neurooncol.

[47] Voltz R, Dalmau J, Posner JB, Rosenfeld MR (1998). T-cell receptor analysis in anti-Hu associated paraneoplastic encephalomyelitis. Neurology.

[48] Tanimura A, Tojyo Y, Turner RJ (2000). Evidence that type I, II, and III inositol 1,4,5-trisphosphate receptors can occur as integral plasma membrane proteins. J Biol Chem.

[49] Lischka FW, Zviman MM, Teeter JH, Restrepo D (1999). Characterization of inositol-1,4,5-trisphosphate-gated channels in the plasma membrane of rat olfactory neurons. Biophys J.

[50] Vermassen E, Parys JB, Mauger JP (2004). Subcellular distribution of the inositol 1,4,5-trisphosphate receptors: functional relevance and molecular determinants. Biol Cell.

[51] Taylor CW, Dellis O (2006). Plasma membrane IP3 receptors. Biochem Soc Trans.

[52] Dellis O, Dedos SG, Tovey SC, Taufiq Ur R, Dubel SJ, Taylor CW (2006). Ca2+ entry through plasma membrane IP3 receptors. Science.

[53] Jarius S, Ruprecht K, Wildemann B, Kuempfel T, Ringelstein M, Geis C, Kleiter I, Kleinschnitz C, Berthele A, Brettschneider J (2012). Contrasting disease patterns in seropositive and seronegative neuromyelitis optica: A multicentre study of 175 patients. J Neuroinflammation.

[54] Mader S, Gredler V, Schanda K, Rostasy K, Dujmovic I, Pfaller K, Lutterotti A, Jarius S, Di Pauli F, Kuenz B (2011). Complement activating antibodies to myelin oligodendrocyte glycoprotein in neuromyelitis optica and related disorders. J Neuroinflammation.

[55] Jarius S, Ruprecht K, Kleiter I, Borisow N, Asgari N, Pitarokoili K, Pache F, Stich O, Beume L, Hümmert MW, et al. MOG-IgG in NMO and related disorders: a multicenter study of 50 patients. Part 1: Frequency, syndrome specificity, influence of disease activity, long-term course, association with AQP4-IgG, and origin. J Neuroinflammation. 2016; doi: 10.1186/s12974-016-0717-1. 10.1186/s12974-016-0717-1 PMC5084340 27788675

[56] Jarius S, Ruprecht K, Kleiter I, Borisow N, Asgari N, Pitarokoili K, Pache F, Stich O, Beume L, Hümmert MW, et al. MOG-IgG in NMO and related disorders: a multicenter study of 50 patients. Part 2: Epidemiology, clinical presentation, radiological and laboratory features, treatment responses, and long-term outcome. J Neuroinflammation. 2016; doi: 10.1186/s12974-016-0718-0. 10.1186/s12974-016-0718-0 PMC5086042 27793206

[57] Jarius S, Kleiter I, Ruprecht K, Asgari N, Pitarokoili K, Borisow N, Hümmert M, Trebst C, Pache F, Winkelmann A, et al. MOG-IgG in NMO and related disorders: a multicenter study of 50 patients. Part 3: Brainstem involvement - frequency, presentation and outcome. J Neuroinflammation. 2016; doi: 10.1186/s12974-016-0719-z. 10.1186/s12974-016-0719-z PMC5088671 27802825

[58] Pache F, Zimmermann H, Mikolajczak J, Schumacher S, Lacheta A, Oertel FC, Bellmann-Strobl J, Jarius S, Wildemann B, Reindl M, et al. MOG-IgG in NMO and related disorders: a multicenter study of 50 patients. Part 4: Afferent visual system damage after optic neuritis in MOG-IgG-seropositive versus AQP4-IgG-seropositive patients. J Neuroinflammation. 2016; doi: 10.1186/s12974-016-0720-6. 10.1186/s12974-016-0720-6 PMC5088645 27802824

[59] Jarius S, Wildemann B (2013). Aquaporin-4 antibodies (NMO-IgG) as a serological marker of neuromyelitis optica: a critical review of the literature. Brain Pathol.

[60] Jarius S, Franciotta D, Bergamaschi R, Wright H, Littleton E, Palace J, Hohlfeld R, Vincent A (2007). NMO-IgG in the diagnosis of neuromyelitis optica. Neurology.

[61] Jarius S, Eichhorn P, Jacobi C, Wildemann B, Wick M, Voltz R (2009). The intrathecal, polyspecific antiviral immune response: specific for MS or a general marker of CNS autoimmunity?. J Neurol Sci.

[62] Jarius S, Franciotta D, Bergamaschi R, Rauer S, Wandinger KP, Petereit HF, Maurer M, Tumani H, Vincent A, Eichhorn P (2008). Polyspecific, antiviral immune response distinguishes multiple sclerosis and neuromyelitis optica. J Neurol Neurosurg Psychiatry.

[63] Jarius S, Franciotta D, Marchioni E, Hohlfeld R, Wildemann B, Voltz R (2006). Intrathecal polyspecific immune response against neurotropic viruses discriminates between multiple sclerosis and acute demyelinating encephalomyelitis. J Neurol.

[64] Hawrylycz MJ, Lein ES, Guillozet-Bongaarts AL, Shen EH, Ng L, Miller JA, van de Lagemaat LN, Smith KA, Ebbert A, Riley ZL (2012). An anatomically comprehensive atlas of the adult human brain transcriptome. Nature.

