# Supplementary material for: Inositol 1,4,5-trisphosphate receptor type 1 autoantibodies in paraneoplastic and non-paraneoplastic peripheral neuropathy
Source: J Neuroinflammation. 2016 Oct 24;13:278. doi: 10.1186/s12974-016-0737-x (PMC5078930; doi:10.1186/s12974-016-0737-x)
Supplement: Additional file 1: Table S1. — List of tumours previously reported in association with Guillain-Barré syndrome. (PDF 94 kb) [file 12974_2016_737_MOESM1_ESM.pdf]

Additional file: Table S1

Jarius S, Ringelstein M, Haas J, Sherysheva II, Komorowski L, Fechner K, Wandinger KP, Albrecht P, Hefter H, Moser A, Neuen-Jacob E, Hartung HP, Wildemann B, Aktas O. Inositol 1,4,5-trisphosphate receptor type 1 autoantibodies in paraneoplastic and non-paraneoplastic peripheral neuropathy. J Neuroinflammation 2016; 13:278; DOI: 10.1186/s12974-016-0737-x.

Tumour type	***References***
Lung cancer	[1-12]
Hodgkin disease	[5, 13-21]
Non-Hodgkin lymphomas and leukaemias	[4, 22-58]
Melanoma	[59, 60]
Breast cancer	[61]
Hepatocellular carcinoma	[62]
Oesophageal carcinoma	[63]
Gastric adenocarcinoma	[64]
Colorectal cancer	[65, 66]
Gall bladder adenocarcinoma	[67-70]
Urinary bladder cancer	[71]
Mesothelioma	[72]
Endometrial carcinoma	[73]
Epidermoid cancer of the tongue	[68]
Parathyroid adenoma	[74]
Pancreatic cancer (including one of neuroendocrine differentiation)	[38, 75]
Disseminated squamous cell carcinoma of unknown origin	[76]

Table S1. List of tumours previously reported in association with Guillain-Barré syndrome

References

1. Naveed S, Okoli K, Hollingsworth J, Kasmani R: **Guillain-Barre syndrome as a paraneoplastic manifestation of small-cell carcinoma of lung.** *South Med J* 2010, **103:**156-158.
2. Watanuki S, Kinoshita K, Oda A, Kobayashi H, Satoh H, Tokuda Y: **Occam's Razor or Hickam's dictum: a paraneoplastic or coincidental occurrence of lung cancer and Guillain-Barre syndrome.** *Intern Med* 2014, **53:**1569-1573.
3. Nokura K, Nagamatsu M, Inagaki T, Yamamoto H, Koga H, Sugimura K, Yoshida M, Hashizume Y: **Acute motor and sensory neuronopathy associated with small-cell lung cancer: a clinicopathological study.** *Neuropathology* 2006, **26:**329-337.
4. Defanti CA, Brambilla A, Erli LC, Tredici G: **[Acute polyradiculoneuritis (Guillain-Barre) associated with the syndrome of inappropriate ADH secretion (Schwartz-Bartter): initial symptom of pulmonary neoplasia].** *Riv Neurobiol* 1984, **30:**303-308.
5. Klingon GH: **The Guillain-Barr'e Syndrome Associated with Cancer.** *Cancer* 1965, **18:**157-163.
6. Tuzun E, Kinay D, Hacohen Y, Aysal F, Vincent A: **Guillain-Barre-like syndrome associated with lung adenocarcinoma and CASPR2 antibodies.** *Muscle Nerve* 2013, **48:**836-837.
7. Ferrufino E, Camarasa A, Chiner E: **Guillain-Barre syndrome as an initial manifestation of small cell lung carcinoma.** *Arch Bronconeumol* 2011, **47:**107-108.
8. Eimil M, Benito-Leon J: **Guillain-Barre-like syndrome heralding small-cell lung cancer.** *Eur J Neurol* 2007, **14:**e15-16.
9. Cicero G, Fulfaro F, Caraceni A, Arcara C, Badalamenti G, Intrivici C, Gebbia N: **A case of Guillain-Barre syndrome in a patient with non small cell lung cancer treated with chemotherapy.** *J Chemother* 2006, **18:**325-327.
10. Togashi K, Shinohara H, Wakabayashi T, Fujita S, Sato K: **[Tetraplegia and respiratory failure due to carcinomatous neuropathy in the early postoperative period of a lung cancer patient: report of a case].** *Kyobu Geka* 2005, **58:**495-498.
11. Lopez Garcia E, Anton Garrido T, Lopez Garcia L, Ramirez Guedes J, Sanabria Gomez F: **[Bronchial carcinoma with a clinical syndrome of Guillain-Barre type of polyradiculitis].** *Rev Clin Esp* 1959, **72:**186-189.

12. Tescola F: **[Case of histiocytic leukemia with initial neurological manifestations of Guillain-Barre syndrome type].** *Clin Pediatr (Bologna)* 1958, **40:**977-989.

13. Hughes CL, Yorio JT, Kovitz C, Oki Y: **Treatment decisions in a man with Hodgkin lymphoma and Guillian-Barre syndrome: a case report.** *J Med Case Rep* 2014, **8:**455.

14. Apjok E, Marosi A, Magyarosy E: **[Guillain-Barre syndrome in patients treated for Hodgkin disease].** *Orv Hetil* 2003, **144:**1039-1040.

15. Maslovsky I, Volchek L, Blumental R, Ducach A, Lugassy G: **Persistent paraneoplastic neurologic syndrome after successful therapy of Hodgkin's disease.** *Eur J Haematol* 2001, **66:**63-65.

16. Correale J, Monteverde DA, Bueri JA, Reich EG: **Peripheral nervous system and spinal cord involvement in lymphoma.** *Acta Neurol Scand* 1991, **83:**45-51.

17. Cameron DG, Howell DA, Hutchison JL: **Acute peripheral neuropathy in Hodgkin's disease; report of a fatal case with histologic features of allergic neuritis.** *Neurology* 1958, **8:**575-577.

18. Lisak RP, Mitchell M, Zweiman B, Orrechio E, Asbury AK: **Guillain-Barre syndrome and Hodgkin's disease: three cases with immunological studies.** *Ann Neurol* 1977, **1:**72-78.

19. Julien J, Vital C, Aupy G, Lagueny A, Darriet D, Brechenmacher C: **Guillain-Barre syndrome and Hodgkin's disease--ultrastructural study of a peripheral nerve.** *J Neurol Sci* 1980, **45:**23-27.

20. Amundson DE, Goodman JC: **Hodgkin's disease in association with Guillain-Barre-Strohl syndrome: case report.** *Mil Med* 1983, **148:**512-513.

21. Cuttner J, Meyer R: **Guillain-Barre syndrome in a patient with Hodgkin's disease.** *Mt Sinai J Med* 1978, **45:**415-417.

22. Vallat JM, De Mascarel HA, Bordessoule D, Jauberteau MO, Tabaraud F, Gelot A, Vallat AV: **Non-Hodgkin malignant lymphomas and peripheral neuropathies--13 cases.** *Brain* 1995, **118 (Pt 5):**1233-1245.

23. Carmona A, Alonso JD, de las Heras M, Navarrete A: **Guillain-Barre syndrome in a patient with diffuse large B-cell lymphoma, and rituximab maintenance therapy. An association beyond anecdotal evidence?** *Clin Transl Oncol* 2006, **8:**764-766.

24. Ozkan A, Taskapilioglu O, Bican A, Ozkocaman V, Ozturk H, Ozkalemkas F, Ali R: **Hairy cell leukemia presenting with Guillain-Barre syndrome.** *Leuk Lymphoma* 2007, **48:**1048-1049.

25. Mailander V, Gleisner B, Blau IW, Thiel E: **Guillain-Barre-Strohl syndrome unraveled as paraneoplastic syndrome of B-cell acute lymphoblastic leukemia in a patient with preceding**

common variable immunodeficiency syndrome with Evans syndrome. *Leuk Lymphoma* 2004, **45:**189-192.

26. Zuk E, Nowacki P, Fabian A: **Guillain-Barre syndrome in patient with Burkitt's lymphoma and type 2 diabetes mellitus.** *Folia Neuropathol* 2001, **39:**281-284.

27. Wada M, Kurita K, Tajima K, Kawanami T, Kato T: **A case of inflammatory demyelinating polyradiculoneuropathy associated with T-cell lymphoma.** *Acta Neurol Scand* 2003, **107:**62-66.

28. Rohmer F, Mengus M, Buchheit F: **[Paraneoplastic neuropathy of the type of the Guillain-Barre syndrome in a patient with solitary myeloma].** *Rev Otoneuroophtalmol* 1962, **34:**97-107.

29. Tzachanis D, Hamdan A, Uhlmann EJ, Joyce RM: **Successful treatment of refractory Guillain-Barre syndrome with alemtuzumab in a patient with chronic lymphocytic leukemia.** *Acta Haematol* 2014, **132:**240-243.

30. Rajeswari B, Krishnan S, Sarada C, Kusumakumary P: **Guillain-Barre syndrome with acute lymphoblastic leukemia.** *Indian Pediatr* 2013, **50:**791-792.

31. Machida H, Shinohara T, Hatakeyama N, Okano Y, Nakano M, Tobiume M, Naruse K, Iwahara Y, Ogushi F: **CD5-positive diffuse large B cell lymphoma infiltrating the central nervous system presenting Guillain-Barre-like syndrome after chemotherapy.** *J Clin Exp Hematop* 2012, **52:**199-204.

32. Brigo F, Balter R, Marradi P, Ferlisi M, Zaccaron A, Fiaschi A, Frasson E, Bertolasi L: **Vincristine-related neuropathy versus acute inflammatory demyelinating polyradiculoneuropathy in children with acute lymphoblastic leukemia.** *J Child Neurol* 2012, **27:**867-874.

33. Polo-Romero FJ, Sanchez-Beteta P, Perona-Buendia P, Perez-Garcia AM: **Guillain-Barre syndrome as first presentation of non-Hodgkin lymphoma.** *Neurologia* 2012, **27:**511-513.

34. Terui K, Takahashi Y, Sasaki S, Kudo K, Kamio T, Ito E: **Guillain-Barre syndrome mimicking acute methotrexate-associated encephalopathy in an adolescent patient with lymphoblastic lymphoma.** *J Pediatr Hematol Oncol* 2010, **32:**615-616.

35. Seffo F, Daw HA: **Non-Hodgkin lymphoma and Guillain-Barre syndrome: a rare association.** *Clin Adv Hematol Oncol* 2010, **8:**201-203.

36. Terenghi F, Ardolino G, Nobile-Orazio E: **Guillain-Barre syndrome after combined CHOP and rituximab therapy in non-Hodgkin lymphoma.** *J Peripher Nerv Syst* 2007, **12:**142-143.

37. Wanschitz J, Dichtl W, Budka H, Loscher WN, Boesch S: **Acute motor and sensory axonal neuropathy in Burkitt-like lymphoma.** *Muscle Nerve* 2006, **34:**494-498.

38. Magne N, Foa C, Castadot P, Otto J, Birtwisle-Peyrottes I, Thyss A: **[Guillain-Barre syndrome and non-Hodgkin's lymphoma. Report of one case and review of literature].** *Rev Med Brux* 2005, **26:**108-111.

39. D'Arena G, Vigliotti ML, Pizza V, Tartarone A, Volpe G, Iodice G, Di Renzo N: **Guillain-Barre syndrome complicating mobilization therapy in a case of B-cell chronic lymphocytic leukemia.** *Leuk Lymphoma* 2004, **45:**1489-1490.

40. Vembu P, Al-Shubaili A, Al-Khuraibet A, Kreze O, Pandita R: **Guillain-Barre syndrome in a case of acute lymphoblastic leukaemia. A case report.** *Med Princ Pract* 2003, **12:**272-275.

41. Aral YZ, Gursel T, Ozturk G, Serdaroglu A: **Guillain-Barre syndrome in a child with acute lymphoblastic leukemia.** *Pediatr Hematol Oncol* 2001, **18:**343-346.

42. Sarmiento MA, Neme D, Fornari MC, Bengio RM: **Guillain-Barre syndrome following 2-chlorodeoxyadenosine treatment for Hairy Cell Leukemia.** *Leuk Lymphoma* 2000, **39:**657-659.

43. Re D, Schwenk A, Hegener P, Bamborschke S, Diehl V, Tesch H: **Guillain-Barre syndrome in a patient with non-Hodgkin's lymphoma.** *Ann Oncol* 2000, **11:**217-220.

44. Geetha N, Hussain BM, Lali VS, Nair MK, Kumar BS: **Guillain-Barre syndrome occurring as a complication of acute nonlymphoblastic leukemia.** *Am J Med* 1999, **107:**100-101.

45. Gutknecht DR: **Guillain-Barre syndrome and SIADH in a patient with chronic lymphocytic leukemia.** *J Am Board Fam Pract* 1998, **11:**237-239.

46. Phan TG, Manoharan A, Pryor D: **Relapse of central nervous system Burkitt's lymphoma presenting as Guillain-Barre syndrome and syndrome of inappropriate ADH secretion.** *Aust N Z J Med* 1998, **28:**223-224.

47. Schwarzer A, Schulze E, Leiblein S, Krahl R, Kubel M, Bartram C, Edelmann J, Sack U, Helbig W: **Guillain Barre syndrome, a possible side effect of buffy coat transfusion and IFN alpha therapy in relapsed CML after bone marrow transplantation.** *Ann Oncol* 1995, **6:**617.

48. Jackson M: **Guillain-Barre syndrome in a patient with chronic lymphocytic leukaemia.** *Postgrad Med J* 1993, **69:**832-833.

49. Rodrigues A, Monteiro A, Viana J, Macedo A, Graca F, Sena A: **Acute non-lymphoblast leukaemia presenting as a Guillain-Barre syndrome.** *J Neurol Neurosurg Psychiatry* 1993, **56:**936-937.

50. Tayal SC, Rowbotham DS, Bansal SK: **Guillain-Barre syndrome in a patient with hairy cell leukaemia.** *J R Soc Med* 1991, **84:**238-239.

51. Kurata H, Hirai M, Miwa A, Murai Y, Mori M: **[B-cell non-Hodgkin's lymphoma associated with lactic acidosis, recurrent acute tumor lysis syndrome, and at the end stage, Guillain-Barre syndrome].** *Nihon Naika Gakkai Zasshi* 1989, **78:**1765-1770.

52. Phanthumchinda K, Intragumtornchai T, Kasantikul V: **Guillain-Barre syndrome and optic neuropathy in acute leukemia.** *Neurology* 1988, **38:**1324-1326.

53. Mactier RA, Khanna R: **Guillain-Barre syndrome in kappa light chain myeloma.** *South Med J* 1987, **80:**1054-1055.

54. Norman M, Elinder G, Finkel Y: **Vincristine neuropathy and a Guillain-Barre syndrome: a case with acute lymphatic leukemia and quadriparesis.** *Eur J Haematol* 1987, **39:**75-76.

55. Taillan B, Pedinielli FJ, Blanc AP: **[Association of Waldenstrom's disease and Guillain Barre syndrome].** *Presse Med* 1985, **14:**844.

56. Sahadevan MG, Raman PT, Hoon RS: **Landry-Guillain-Barre syndrome complicating lymphosarcoma. A case report.** *J Assoc Physicians India* 1969, **17:**215-216.

57. Powles RL, Malpas JS: **Guillain-Barre syndrome associated with chronic lymphatic leukaemia.** *Br Med J* 1967, **3:**286-287.

58. Carcassi A, Gentili M, Pianigiani A: **[Guillain-Barre syndrome secondary to systemic reticulosarcomatosis].** *Atti Accad Fisiocrit Siena Med Fis* 1964, **13:**637-650.

59. Kraft Rovere R, Pires de Souza ME, Fernanda Hilgert S, Rodrigues Chamse Ddine Y, Silva de Lima A: **Melanoma metastasis to the gastric mucosa preceded by guillain-barre as a paraneoplastic syndrome.** *Gastrointest Cancer Res* 2013, **6:**150-151.

60. Ben Simon GJ, McCann JD, Barth N, Goldberg RA, Glasgow BJ, Straatsma BR: **Partial resolution of acute ascending motor polyneuropathy after enucleation of an eye with metastatic melanoma.** *Br J Ophthalmol* 2004, **88:**847.

61. Estrada Perez V, Arroyo Serrano S, Garcia Gonzalez C, Garcia Asensio JA, Gutierrez Marcos FM: **[Breast cancer associated with hypercalcemia and Guillain-Barre syndrome].** *Rev Clin Esp* 1990, **187:**209-210.

62. Camdessanche JP, Antoine JC, Honnorat J, Vial C, Petiot P, Convers P, Michel D: **Paraneoplastic peripheral neuropathy associated with anti-Hu antibodies. A clinical and electrophysiological study of 20 patients.** *Brain* 2002, **125:**166-175.

63. Zilli T, Allal AS: **Guillain-Barre syndrome as an atypical manifestation of an esophageal carcinoma.** *Neurol Sci* 2011, **32:**151-153.

64. Tola-Arribas MA, Canibano-Gonzalez MA: **[Guillain-Barre syndrome associated with gastric adenocarcinoma. Paraneoplastic origin or coincidence?].** *Rev Neurol* 2001, **33:**797-798.

65. Christodoulou C, Anastasopoulos D, Visvikis A, Mellou S, Detsi I, Tsiakalos G, Pateli A, Klouvas G, Papadimitriou A, Skarlos DV: **Guillain-Barre syndrome in a patient with metastatic colon cancer receiving oxaliplatin-based chemotherapy.** *Anticancer Drugs* 2004, **15:**997-999.

66. Vatandoust S, Joshi R, Price TJ: **Guillain-Barre syndrome in colorectal cancer.** *Asia Pac J Clin Oncol* 2012, **8:**205-208.

67. Yoon JY, Nam TS, Kim MK, Hwang JE, Shim HJ, Cho SH, Chung IJ, Bae WK: **Acute inflammatory demyelinating polyradiculoneuropathy in a patient receiving oxaliplatin-based chemotherapy.** *Asia Pac J Clin Oncol* 2012, **8:**201-204.

68. Antoine JC, Mosnier JF, Absi L, Convers P, Honnorat J, Michel D: **Carcinoma associated paraneoplastic peripheral neuropathies in patients with and without anti-onconeural antibodies.** *J Neurol Neurosurg Psychiatry* 1999, **67:**7-14.

69. Phan TG, Hersch M, Zagami AS: **Guillain-Barre syndrome and adenocarcinoma of the gall bladder: a paraneoplastic phenomenon?** *Muscle Nerve* 1999, **22:**141-142.

70. Szoeke T, Hzffner A, Komar J: **[Cancer of the Gallbladder Causing Guillain-Barre Syndrome].** *Ideggyogy Sz* 1963, **16:**321-327.

71. Lagrange E, Veran O, Besson G: **Pure motor relapsing Guillain-Barre syndrome associated with anti-GM1 antibodies revealing urinary bladder cancer.** *Eur J Neurol* 2007, **14:**e7.

72. Sights WP, Jr., Kam CC, Almond CF, Logue JT: **The Guillain-Barre syndrome associated with a mesothelioma. Case report.** *Mo Med* 1968, **65:**897-899 passim.

73. Tho LM, O'Leary CP, Horrocks I, Al-Ani A, Reed NS: **Guillain-Barre syndrome occurring after adjuvant chemo-radiotherapy for endometrial cancer.** *Gynecol Oncol* 2006, **100:**615-617.

74. Vallat JM, Poumier C, Dumas M, Gastinne H, Gobeaux R: **Guillain-Barre syndrome and parathyroid adenoma.** *Arch Neurol* 1982, **39:**322.

75. Yu TC, Omundsen M, Rahman H: **Pancreatic neuroendocrine tumour occurring with Guillian-Barre syndrome.** *ANZ J Surg* 2010, **80:**297.

76. Navani V, Webster D, Williams SK, Agranoff D: **Guillain-Barre syndrome as a paraneoplastic manifestation of disseminated squamous cell carcinoma.** *BMJ Case Rep* 2013, **2013**.
